# In the Era of Cardiovascular–Kidney–Metabolic Syndrome in Cardio-Oncology: From Pathogenesis to Prevention and Therapy

**DOI:** 10.3390/cancers17071169

**Published:** 2025-03-30

**Authors:** Vincenzo Quagliariello, Massimiliano Berretta, Irma Bisceglia, Ilaria Giacobbe, Martina Iovine, Matteo Barbato, Carlo Maurea, Maria Laura Canale, Andrea Paccone, Alessandro Inno, Marino Scherillo, Stefano Oliva, Christian Cadeddu Dessalvi, Alfredo Mauriello, Celeste Fonderico, Anna Chiara Maratea, Domenico Gabrielli, Nicola Maurea

**Affiliations:** 1 Division of Cardiology, Istituto Nazionale Tumori-IRCCS-Fondazione G. Pascale, 80131 Napoli, Italy; 2 Department of Clinical and Experimental Medicine, University of Messina, 98125 Messina, Italy; 3 Servizi Cardiologici Integrati, Dipartimento Cardio-Toraco-Vascolare, Azienda Ospedaliera San Camillo Forlanini, 00148 Rome, Italy; 4 ASL NA1, UOC Neurology and Stroke Unit, Ospedale del Mare, 23807 Naples, Italy; 5 U.O.C. Cardiologia, Ospedale Versilia, 55041 Lido di Camaiore, Italy; 6 Medical Oncology, IRCCS Ospedale Sacro Cuore Don Calabria, 37024 Negrar di Valpolicella, Italy; 7 Cardiologia Interventistica e UTIC, A.O. San Pio, Presidio Ospedaliero Gaetano Rummo, 82100 Benevento, Italy; 8 Cardio-Oncology Unit, IRCCS Istituto Tumori, “Giovanni Paolo II”, 70124 Bari, Italy; 9 Department of Medical Sciences and Public Health, University of Cagliari, 09123 Cagliari, Italy; 10 U.O.C. Cardiologia, Dipartimento Cardio-Toraco-Vascolare, Azienda Ospedaliera San Camillo Forlanini, 00152 Rome, Italy

**Keywords:** metabolic syndrome, kidney diseases, cancer, cardio-oncology, cardiotoxicity, metabolism, PCSK9, obesity

## Abstract

Cardiovascular–kidney–metabolic (CKM) syndrome represents a complex interplay between cardiovascular disease (CVD), chronic kidney disease (CKD), and metabolic disorders, significantly impacting cancer patients. The presence of CKM syndrome in cancer patients not only worsens their prognosis but also increases the risk of major adverse cardiovascular events (MACE), reduces quality of life (QoL), and affects overall survival (OS). Furthermore, several anticancer therapies, including anthracyclines, tyrosine kinase inhibitors, immune checkpoint inhibitors, and hormonal treatments, can exacerbate CKM syndrome by inducing cardiotoxicity, nephrotoxicity, and metabolic dysregulation. This review explores the pathophysiology of CKM syndrome in cancer patients and highlights emerging therapeutic strategies to mitigate its impact. We discuss the role of novel pharmacological interventions, including sodium-glucose cotransporter-2 inhibitors (SGLT2i), proprotein convertase subtilisin/kexin type 9 inhibitors (PCSK9i), and soluble guanylate cyclase (sGC) activators, as well as dietary and lifestyle interventions. Optimizing the management of CKM syndrome in cancer patients is crucial to improving OS, enhancing QoL, and reducing MACE. By integrating cardiometabolic therapies into oncologic care, we can create a more comprehensive treatment approach that reduces the burden of cardiovascular and renal complications in this vulnerable population. Further research is needed to establish personalized strategies for CKM syndrome prevention and treatment in cancer patients.

## Simple Summary

Cardiovascular–kidney–metabolic (CKM) syndrome is a significant yet underrecognized condition in cancer patients, contributing to increased morbidity, reduced quality of life (QoL), and lower overall survival (OS). CKM syndrome arises from the interconnected pathophysiology of cardiovascular disease (CVD), chronic kidney disease (CKD), and metabolic dysfunction, which are further exacerbated by cancer therapies. Anticancer treatments such as anthracyclines, immune checkpoint inhibitors, tyrosine kinase inhibitors, and hormonal therapies can accelerate CKM progression by inducing cardiotoxicity, nephrotoxicity, and metabolic disturbances. To address these challenges, a multidisciplinary approach incorporating novel therapeutic strategies is essential. Emerging treatments include SGLT2i for their reno-cardiometabolic benefits, PCSK9i to reduce atherosclerotic cardiovascular disease (ASCVD) risk, and soluble guanylate cyclase activators for endothelial and cardiac function improvement. Additionally, dietary and lifestyle interventions play a crucial role in reduction of metabolic dysfunction and enhancing overall patient outcomes. By integrating these cardiometabolic therapies into oncology care, we can potentially improve OS, enhance QoL and reduce major adverse cardiovascular events (MACE) in cancer patients with CKM syndrome. Further clinical research is needed to optimize personalized strategies for CKM prevention and treatment in this high-risk population.

## 1. Introduction

Cardio-oncology has emerged as a critical field due to the growing population of cancer survivors, many of whom are at an increased risk for cardiovascular diseases (CVD) as a consequence of cancer therapies, including chemotherapy, immune checkpoint inhibitors, and radiation [1]. These treatments can induce direct and indirect damages to cardiomyocytes and endothelial cells, leading to long-term sequelae such as heart failure, arrhythmias, and accelerated atherosclerosis [2]. Furthermore, the rising prevalence of obesity and metabolic syndrome in cancer survivors exacerbates this risk. Obesity and metabolic syndrome, which involves a cluster of diseases including insulin resistance, hypertension, dyslipidemia, and central adiposity, increases the risk of cardiovascular–kidney–metabolic (CKM) syndrome. In brief, CKM syndrome refers to the interconnected dysfunction of the cardiovascular system, kidneys, and metabolic pathways, forming a vicious cycle of disease progression [3]. The pathogenesis of CKM syndrome is complex, involving interactions between hemodynamic, inflammatory, neurohormonal, and metabolic pathways [4]. Dysregulation of these systems often occurs in parallel, leading to mutual exacerbation of each organ system. For example, impaired renal function can exacerbate cardiovascular disease by promoting fluid retention, hypertension, and increased cardiovascular workload [5]. Conversely, cardiovascular dysfunction can worsen renal outcomes by reducing renal perfusion and inducing glomerular damage. Additionally, metabolic abnormalities, such as insulin resistance and dyslipidemia, contribute to the development and progression of both cardiovascular and kidney diseases, creating a feedback loop that accelerates disease progression across all three organ systems [6]. As illustrated in Table 1, the progression of metabolic and cardiovascular dysfunction of CKM can be summarized in five stages, based on clinical, pathophysiological, and cardiovascular risk profiles [7]: Stage 0 represents a physiological baseline, characterized by the absence of metabolic, renal, or cardiovascular dysfunction, with preserved metabolic and hemodynamic homeostasis, corresponding to a low CVD risk. Stage 1 is defined by the presence of central obesity and early metabolic perturbations, driven by adipose tissue dysfunction, the onset of mild insulin resistance, and a state of chronic low-grade inflammation, collectively conferring a borderline CVD risk. Stage 2 involves metabolic syndrome and/or early renal impairment, with hallmark features including hypertension, dyslipidemia, hyperglycemia, and increased renal stress, indicating an intermediate CVD risk. Stage 3 is characterized by subclinical cardiovascular disease associated with metabolic and renal dysfunction, evidenced by endothelial dysfunction, left ventricular remodeling, and increased arterial stiffness, signifying a high CVD risk. Stage 4 represents the late phase of this *continuum*, wherein overt cardiovascular disease coexists with advanced metabolic and renal dysfunction, typified by the presence of atherosclerosis, heart failure, and progressive chronic kidney disease, culminating in a very high CVD risk [8,9] (Table 1). This classification framework underscores the progressive and interrelated nature of metabolic, renal, and cardiovascular dysfunction, emphasizing the escalating pathophysiological burden and corresponding risk stratification across disease stages.

Cancer patients, particularly survivors, are at heightened risk for developing CKM syndrome due to the systemic toxicities associated with various oncological therapies [10]. Immune checkpoint inhibitors (ICIs), chemotherapy, targeted therapies, and radiotherapy, while critical in cancer treatment, can all contribute to the pathogenesis of CKM syndrome [11]. Notably, ICIs have been linked to immune-mediated adverse effects, including systemic inflammation and endothelial dysfunction, which can exacerbate cardiovascular and renal damages [12]. Chemotherapeutic agents, particularly those with known cardiotoxic profiles, increase the risk of myocardial dysfunction, arrhythmias, and heart failure. Targeted therapies, while designed to target specific molecular pathways, can induce metabolic diseases, including insulin resistance, dyslipidemia, and hypertension, further increasing cardiovascular and renal risk [13].

Radiotherapy, especially when applied to thoracic or abdominal regions, can induce direct vascular and renal damage, leading to fibrosis, endothelial dysfunction, and renal impairment [14]. Cancer survivors face a significantly increased risk of developing cardiovascular and renal diseases, with these complications emerging years or even decades after completing cancer therapy. Epidemiological studies indicate that cancer survivors have a 1.3- to 3-fold higher risk of cardiovascular disease (CVD) compared to the general population, depending on cancer type and treatment exposure [4]. For instance, in large cohort studies, the incidence of heart failure among breast cancer survivors treated with anthracyclines or trastuzumab reaches 15–20%, while survivors of hematological malignancies undergoing hematopoietic stem cell transplantation (HSCT) show a 5- to 10-fold increased risk of cardiovascular events compared to age-matched controls [13].

The pathophysiology underlying these long-term complications is multifactorial: in fact, cancer therapies, particularly anthracyclines, tyrosine kinase inhibitors, ICIs, and radiotherapy, induce endothelial dysfunction, mitochondrial damages, and oxidative stress, all of which contribute to accelerated atherosclerosis and cardiac remodeling [14]. Chronic inflammation, metabolic dysregulation, and persistent immune activation further exacerbate these processes.

Moreover, renal complications are also a growing concern, with long-term cancer survivors exhibiting a two-fold higher risk of CKD [15]. Nephrotoxic chemotherapy agents such as cisplatin and ifosfamide, as well as ICIs, contribute to glomerular injury, tubular dysfunction, and progressive fibrosis [15]. Furthermore, metabolic syndrome, characterized by insulin resistance, hypertension, obesity, and dyslipidemia, acts as a key driver of both cardiovascular and renal dysfunction in this population. Moreover, the available studies indicate that the prevalence of CKM-related conditions (obesity, metabolic syndrome, diabetes, and CKM) ranges from 25% to 40% in the general population [15]. In cancer patients, these rates are significantly higher due to shared risk factors and cancer therapy-induced metabolic diseases. In cancer patients, CKM syndrome prevalence varies based on cancer type and treatment exposure: in more detail, large cohort studies report that up to 50% of breast cancer survivors develop metabolic syndrome post-therapy, especially after endocrine therapy [8]; moreover, patients with hematologic malignancies undergoing hematopoietic stem cell transplantation (HSCT) show a 30–50% risk of developing metabolic syndrome post-transplant. Instead, among prostate cancer patients on androgen deprivation therapy (ADT), the incidence of new-onset diabetes reaches 20–25% over 5 years [15]. For kidney dysfunction, platinum-based chemotherapies (e.g., cisplatin) are associated with 30–40% rates of CKD post-treatment [15].

Notably, these data suggest that CKM syndrome is not only a pre-existing risk in oncology patients but is also substantially exacerbated by cancer therapy. Therefore, the interplay between CKM syndrome and long-term survivorship necessitates early risk stratification and preventive strategies.

The integration of cardiometabolic screening and reno-protective therapies (e.g., SGLT2 inhibitors, GLP-1 receptor agonists, RAAS inhibitors) into survivorship care plans may mitigate long-term morbidity. Future research should focus on personalized strategies for cardiovascular and renal risk reduction in cancer survivors, considering both genetic susceptibility and treatment-specific toxicities.

The cumulative effects of these therapies, associated with pre-existing cancer-related risk factors, induce a multifactorial pathophysiological environment where cardiovascular, renal, and metabolic dysfunctions are interconnected.

Understanding the multifactorial interactions in CKM syndrome is essential for developing effective therapeutic strategies to break this cycle and mitigate long-term morbidity and mortality [15].

## 2. Methods

A narrative search of the Medline and EMBASE databases was performed to identify relevant papers reporting original research (involving RCTs, cohort studies, meta-analyses) on the effects of CKM in cancer patients of the past 10 years. Clinical studies on the effects of CKM in cardiology and cardio-oncology were included if they met the following criteria:

- Published in English with an available abstract
- Addressed at least one of the following topics: CKM, metabolic syndrome, SGLT2 inhibitors (SGLT2i), PCSK9 inhibitors (PCSK9i), soluble guanylate cyclase activators (SGCa), GLP-1 receptor agonists, diet, inflammation, diabetes, cardiotoxicity, cardioprotection, and cancer

Studies were excluded if they were reviews, case reports, lacked immunomodulation activity endpoints, or were conducted in surgical settings (Table 2). We qualitatively synthesized the findings from the selected studies to provide an overview of the effects of CKM in cancer and cardiology. The search strategy incorporated Boolean operators (AND, OR) without truncation (*) to optimize retrieval of relevant articles. The exact search strings used in each database are detailed in Table 2. The databases were last accessed on 20 February 2025.

## 3. CMK Syndrome: Pathogenesis and Clinical Implications

**Figure 1:**
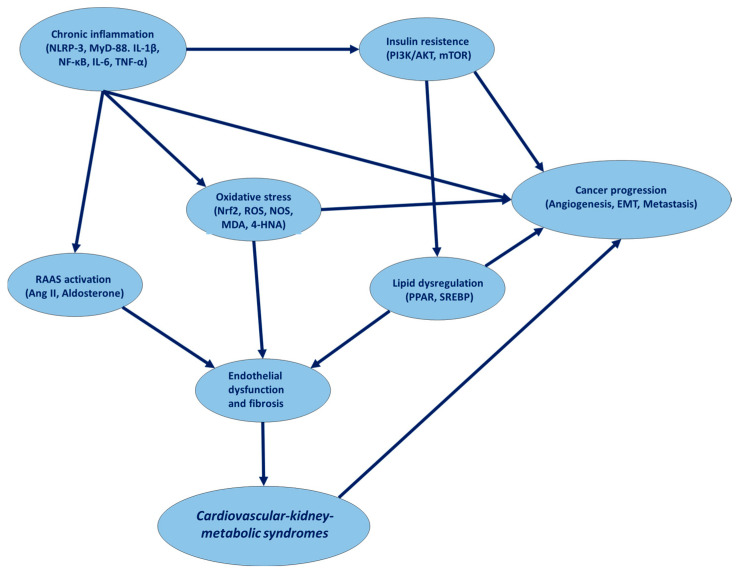
Overall description of CV risk, kidney and metabolic dysfunction, cancer-related toxicity, survival, inflammation, and therapeutic challenges during CKM.

In brief, CKM syndrome is a systemic disorder defined by the coexistence of metabolic abnormalities (such as obesity, insulin resistance, and dyslipidemia), renal impairment, and cardiovascular dysfunction [16]. These interrelated pathologies contribute to a continuum of disease progression, ultimately increasing morbidity and mortality associated with cardiovascular and renal complications [17]. As reported in introduction, CKM pathogenesis involves hemodynamic, inflammatory, neurohormonal, and metabolic pathways (Figure 1). The intricate interplay between cardiovascular, renal, and metabolic dysfunction results in a progressive and self-reinforcing disease process [18]. Hemodynamic overload, chronic inflammation, and dysregulated neurohormonal activity drive the onset and progression of cardiovascular and kidney disease, while metabolic derangements—including insulin resistance and dyslipidemia—further compound these pathological mechanisms [18]. As a result, CKM syndrome is linked to heightened morbidity and mortality, with an increased likelihood of adverse outcomes such as heart failure, chronic kidney disease, and diabetes [19].

### 3.1. Neurohormonal Activation

In CKM syndrome, neurohormonal activation serves as a central mechanism of disease progression, involving a dynamic interaction between the renin–angiotensin–aldosterone system (RAAS), the sympathetic nervous system (SNS), and natriuretic peptides [20]. These systems, when dysregulated, create a biochemical environment that perpetuates organ dysfunction across the heart, kidneys, and metabolic systems. The renin–angiotensin–aldosterone system (RAAS) is a key regulatory mechanism for blood pressure and fluid balance; however, its overactivation in CKM syndrome triggers a cascade of detrimental effects [21]. In response to perceived hypoperfusion or sodium depletion, the kidneys release renin, which catalyzes the conversion of angiotensinogen (from the liver) into angiotensin I. Angiotensin I is then converted to angiotensin II, primarily in the lungs, by angiotensin-converting enzyme (ACE) [22]. Angiotensin II, a potent vasoconstrictor, increases systemic vascular resistance, contributing to hypertension. It also stimulates cardiac hypertrophy and fibrosis through activation of angiotensin II type 1 (AT1) receptors in the heart and vasculature. Moreover, angiotensin II induces aldosterone secretion from the adrenal cortex, leading to sodium retention and potassium excretion in the kidneys [22]. This results in fluid retention, further elevating blood pressure and increasing the mechanical burden on the heart while accelerating renal damage due to high glomerular pressure. Simultaneously, the sympathetic nervous system (SNS) becomes hyperactivated in CKM, compounding the pathological effects [23]. Chronic SNS overactivation leads to excessive release of norepinephrine which bind to adrenergic receptors in the heart, kidneys, and vasculature [24]. In the heart, this results in increased heart rate and myocardial contractility, which are initially compensatory but ultimately maladaptive, promoting arrhythmias, hypertrophy and heart failure. In the kidneys, sympathetic activation reduces renal perfusion, causes afferent arteriolar vasoconstriction, and diminishes glomerular filtration rate (GFR) [25]. Furthermore, SNS stimulation augments renin release, reinforcing RAAS activation. In the vasculature, norepinephrine reduces nitric oxide bioavailability, promoting endothelial dysfunction and contributing to the development of hypertension (Figure 1) [26]. Natriuretic peptides, including atrial natriuretic peptide (ANP) and brain natriuretic peptide (BNP), counteract the effects of the RAAS by promoting natriuresis, diuresis, and vasodilation [27]. However, in CKM syndrome, this counter-regulatory system is often overwhelmed or desensitized, contributing to persistent fluid overload and hypertension. Chronic elevation of natriuretic peptides, driven by ongoing volume overload, leads to downregulation of their receptors, reducing their effectiveness and blunting their protective actions [28]. This results in further sodium and fluid retention, exacerbating the burden on the cardiovascular and renal systems. Moreover, these neurohormonal diseases lead to the production of ROS and pro-inflammatory cytokines, fueling oxidative stress and inflammation [29]. The overactivation of RAAS and SNS, combined with the diminished function of natriuretic peptides, creates a self-perpetuating cycle that accelerates cardiovascular and renal injury, while also intensifying metabolic dysfunction. This cascade of events facilitates the full progression of CKM syndrome [30] (Figure 1).

### 3.2. Chronic Inflammation

Chronic inflammation plays a central role in the pathogenesis of CKM syndrome, acting as a bridge between metabolic dysfunction and the structural and functional decline of the heart and kidneys [31]. The inflammatory state in CKM arises from a combination of metabolic imbalances, endothelial dysfunction, and tissue injury, driving a continuous cycle of immune activation and biochemical damage [32].

Notably, the metabolic alterations characteristic of CKM, such as hyperglycemia, insulin resistance and dyslipidemia, provide a fertile ground for the initiation of inflammation [33]. Hyperglycemia triggers the formation of advanced glycation end products (AGEs), which accumulate in tissues and interact with receptors for AGEs (RAGE) on immune and endothelial cells [34]. This interaction activates nuclear factor-kappa B (NF-κB) pathway, leading to the production of pro-inflammatory cytokines like tumor necrosis factor-alpha (TNF-α), interleukin-6 (IL-6), and interleukin-1β (IL-1β) [35]. These cytokines not only exacerbate tissue damage but also recruit immune cells, such as macrophages and T cells, to sites of injury, perpetuating the inflammatory response (Figure 1).

Simultaneously, oxidative stress serves as both a consequence and amplifier of inflammation in CKM. The excessive production of ROS by mitochondria, nicotinamide adenine dinucleotide phosphate (NADPH) oxidase, and uncoupled nitric oxide synthase (NOS) overwhelms antioxidant defenses [36]. ROS interact with lipids, proteins, and DNA, causing cellular dysfunction and further promoting the activation of inflammatory pathways. For example, ROS can enhance NF-κB signaling and stimulate the release of pro-inflammatory cytokines, creating a feedback loop that sustains oxidative stress and inflammation [37].

The kidneys and cardiovascular system are particularly vulnerable to these inflammatory processes. In the kidneys, the infiltration of macrophages and T cells into the renal parenchyma leads to the release of proteolytic enzymes and cytokines that damage the glomeruli and tubulointerstitial compartments [38]. This contributes to the progression of glomerulosclerosis and tubulointerstitial fibrosis, impairing renal filtration and exacerbating proteinuria. In the heart, inflammatory cells and cytokines promote myocardial fibrosis and apoptosis, weakening cardiac function and paving the way for heart failure [39].

Endothelial dysfunction is another key feature of inflammation in CKM [40]. The endothelium, under normal conditions, maintains vascular homeostasis by producing vasodilators like nitric oxide (NO) and anti-inflammatory factors [41]. However, chronic inflammation disrupts this balance, as cytokines and ROS reduce NO bioavailability and increase the expression of adhesion molecules like intercellular adhesion molecule-1 (ICAM-1) and vascular cell adhesion molecule-1 (VCAM-1). These adhesion molecules facilitate the attachment and migration of leukocytes into the vessel wall, leading to the development of atherosclerosis [42]; over time, these vascular changes contribute to hypertension and reduced perfusion of the kidneys and other organs.

The systemic nature of inflammation in CKM also has significant metabolic consequences. TNF-α and IL-6 interfere with insulin signaling pathways, particularly by inhibiting the insulin receptor substrate (IRS) and downstream targets like phosphatidylinositol 3-kinase (PI3K) and Akt [43]. This promotes insulin resistance, which exacerbates hyperglycemia and perpetuates the inflammatory state. Additionally, chronic inflammation alters adipocyte function, leading to the release of free fatty acids and adipokines like leptin and resistin, which further aggravate insulin resistance and vascular inflammation [44]. Therefore, chronic inflammation in CKM syndrome is a self-sustaining process fueled by metabolic imbalances, oxidative stress, and endothelial dysfunction [45]. Through the release of cytokines, ROS, and other inflammatory mediators, this state drives a cycle of tissue injury and immune activation, leading to progressive dysfunction of the cardiovascular and renal systems while worsening metabolic dysregulation [46].

### 3.3. Hemodynamic Abnormalities

Hemodynamic abnormalities are a pivotal component in the pathogenesis of CKM syndrome, intertwining mechanical and biochemical stressors that dysregulate normal cardiovascular and renal function [47]. These abnormalities primarily manifest as sustained hypertension, volume overload, and altered perfusion dynamics, creating a feedback loop of progressive organ dysfunction (Table 3).

Hypertension, a hallmark feature of CKM, arises from a combination of neurohormonal overactivation, endothelial dysfunction, and renal impairments. Persistent activation RAAS increases systemic vascular resistance through angiotensin II [48]. On a cellular level, angiotensin II interacts with angiotensin II type 1 (AT1) receptors in vascular smooth muscle cells, triggering calcium influx via L-type calcium channels. This enhances smooth muscle contraction, leading to narrowed blood vessels and increased afterload on the heart [49]. Over time, this chronic pressure overload induces left ventricular hypertrophy and myocardial remodeling, compromising cardiac efficiency and contributing to diastolic dysfunction (Table 3).

In the kidneys, hypertension exacerbates intraglomerular pressure, a direct consequence of both systemic hypertension and the effects of angiotensin II on renal arterioles [50]. Angiotensin II preferentially constricts the efferent arteriole, maintaining glomerular filtration in the short term but increasing the mechanical stress on glomerular capillaries. This heightened pressure accelerates glomerulosclerosis and the loss of functional nephrons, reducing renal filtration capacity and leading to progressive kidney injury [51]. Proteinuria often develops as a result of glomerular barrier dysfunction, further aggravating tubular inflammation and fibrosis [52].

Volume overload is another critical hemodynamic disturbance in CKM, stemming from sodium and water retention due to impaired renal function. The kidneys, in response to reduced effective arterial blood volume, increase sodium reabsorption via the activation of RAAS and sympathetic nervous system pathways [53]. Aldosterone acts on mineralocorticoid receptors in the distal nephron, upregulating the expression of sodium-potassium channels and sodium–chloride co-transporters, leading to enhanced sodium and water retention. This fluid accumulation raises venous pressures, contributing to the development of peripheral edema, pulmonary congestion, and increased preload on the heart [54]. The ensuing elevation in cardiac filling pressures exacerbates heart failure, creating a vicious cycle of volume retention and cardiac dysfunction.

The interplay between reduced renal perfusion and cardiac output further compounds hemodynamic disturbances. In CKM, a failing heart often struggles to maintain adequate cardiac output, reducing renal blood flow and triggering compensatory mechanisms such as RAAS activation and sympathetic nervous system stimulation [55]. Paradoxically, these compensatory responses worsen hypertension and fluid retention, perpetuating renal and cardiac decline. In the context of reduced perfusion, hypoxic injury to renal tubular cells occurs, leading to the release of DAMPS (damage-associated molecular patterns) that activate inflammatory pathways and contribute to chronic kidney injury [56].

On the vascular level, endothelial dysfunction is a key mediator of hemodynamic abnormalities. In CKM, endothelial cells exhibit reduced nitric oxide (NO) bioavailability due to oxidative stress and the downregulation of endothelial nitric oxide synthase (eNOS) [57]. NO, a critical vasodilator, normally opposes the vasoconstrictive effects of angiotensin II and catecholamines. Its reduction leads to unopposed vasoconstriction, heightened vascular resistance, and further elevation of blood pressure [58].

Additionally, endothelial dysfunction promotes vascular stiffness through the deposition of collagen and other extracellular matrix components, increasing pulse wave velocity and contributing to isolated systolic hypertension. Therefore, hemodynamic abnormalities in CKM syndrome represent a dynamic interplay of mechanical and biochemical dysfunctions [59]. Hypertension, driven by RAAS overactivation and endothelial dysfunction, imposes structural and functional stress on the heart and kidneys. Volume overload and reduced perfusion amplify these effects, creating a self-reinforcing cycle that perpetuates organ damage and underpins the progression of CKM syndrome [60] (Table 3).

### 3.4. Metabolic Dysregulation

Metabolic dysregulation is another key element in the pathogenesis of CKM syndrome, involving insulin resistance, hyperglycemia, dyslipidemia, and systemic inflammation. These metabolic disturbances are interconnected and contribute to the structural and functional deterioration of both the cardiovascular and renal systems [61].

Insulin resistance is a fundamental driver of metabolic dysfunction in CKM. In brief, it occurs when cells in peripheral tissues, such as skeletal muscle, adipose tissue, and the liver, fail to respond adequately to insulin signaling [62]. Under normal physiological conditions, insulin binds to its receptor, triggering autophosphorylation of the insulin receptor and activation of downstream signaling pathways, including the phosphoinositide 3-kinase (PI3K)-Akt pathway [63]. This promotes glucose uptake by translocating glucose transporter type 4 (GLUT4) to the cell membrane, glycogen synthesis, and lipid storage. In CKM, chronic inflammation and oxidative stress interfere with these pathways. Pro-inflammatory cytokines like tumor necrosis factor-alpha (TNF-α) and interleukin-6 (IL-6) activate serine kinases such as c-Jun N-terminal kinase (JNK) and inhibitor of nuclear factor kappa-B kinase (IKK). These kinases phosphorylate insulin receptor substrate (IRS) proteins on serine residues, impairing their ability to propagate insulin signaling. As a result, glucose uptake is diminished, leading to hyperglycemia [64].

Hyperglycemia, in turn, exerts toxic effects on tissues through several mechanisms. One major pathway is the overproduction of ROS during glucose metabolism. In the mitochondria, excessive glucose increases the flow of electrons through the electron transport chain, leading to leakage of electrons and the generation of superoxide anions. ROS damage cellular components, including lipids, proteins, and DNA, contributing to oxidative stress and tissue injury [65]. Hyperglycemia also drives the formation of AGEs, which accumulate in tissues and bind to their receptor (RAGE) on endothelial cells and immune cells. This interaction triggers the activation of NF-κB that upregulates the expression of pro-inflammatory cytokines, adhesion molecules, and ROS-producing enzymes, perpetuating inflammation and vascular damages [66].

Dyslipidemia is another critical aspect of metabolic dysregulation in CKM. Insulin resistance reduces normal lipid metabolism by impairing insulin’s inhibitory effects on hormone-sensitive lipase in adipocytes. This leads to increased lipolysis and the release of free fatty acids (FFAs) into the circulation [67]. In brief, excess FFAs are taken up by the liver, where they promote triglyceride synthesis and the production of very-low-density lipoproteins (VLDLs) [68]. High levels of VLDLs and low-density lipoproteins (LDLs), coupled with reduced levels of high-density lipoproteins (HDLs), create a pro-atherogenic lipid profile. In the vascular system, oxidized LDL particles are taken up by macrophages via scavenger receptors, leading to foam cell formation and the development of atherosclerotic plaques [69]. Additionally, FFAs can accumulate in non-adipose tissues, such as the myocardium and kidneys, causing lipotoxicity. This induces cellular stress and apoptosis, contributing to cardiac and renal dysfunction.

Kidneys are particularly vulnerable to the effects of metabolic dysregulation; in fact, hyperglycemia increases the renal glucose load, leading to heightened glucose reabsorption by sodium-glucose co-transporters (SGLTs) in the proximal tubules [70]. This not only exacerbates hyperglycemia but also reduces sodium delivery to the macula densa, impairing tubuloglomerular feedback and sustaining activation of RAAS pathway. The combination of glomerular hyperfiltration, oxidative stress, and inflammatory cytokines accelerates the development of diabetic nephropathy, characterized by glomerulosclerosis, proteinuria, and progressive loss of renal function [71]. Therefore, metabolic dysregulation in CKM syndrome is characterized by a complex interplay of insulin resistance, hyperglycemia, dyslipidemia, and inflammation [72]. These processes create a biochemical environment that damages vascular, cardiac, and renal tissues, perpetuating a cycle of metabolic and organ dysfunction that underpins the progression of CKM syndrome [73].

### 3.5. Fibrosis

Fibrosis and structural damage represent the culmination of chronic pathological processes in CKM syndrome. This structural remodeling involves excessive deposition of ECM components such as collagen and fibronectin, leading to impaired organ function and irreversible tissue damage [74].

In the myocardium, fibrosis is a hallmark of heart failure and is primarily mediated by cardiac fibroblasts. Under pathological conditions, such as chronic pressure overload or ischemic injury, fibroblasts are activated by signaling molecules like transforming growth factor-beta (TGF-β) and angiotensin II. TGF-β, a key profibrotic cytokine, binds to its receptors on fibroblasts, initiating Smad-dependent and Smad-independent pathways that drive the transcription of ECM genes [75]. Angiotensin II contributes by interacting with AT1 receptors, activating downstream kinases such as mitogen-activated protein kinases (MAPKs) and protein kinase C (PKC), which further amplify fibroblast activation. The resultant overproduction of ECM leads to interstitial and perivascular fibrosis, increasing myocardial stiffness and reducing ventricular compliance. This diastolic dysfunction ultimately impairs the heart’s ability to fill properly, exacerbating heart failure [76].

In the kidneys, fibrosis occurs through similar mechanisms but has unique features tied to renal structure. Chronic injury, such as that caused by hypertension, proteinuria, or hyperglycemia, leads to tubular epithelial cell damage and apoptosis. These injured tubular cells release DAMPs that activate nearby fibroblasts and recruit immune cells [77]. Macrophages and other inflammatory cells infiltrate the interstitium, secreting cytokines and growth factors, including TGF-β, platelet-derived growth factor (PDGF), and connective tissue growth factor (CTGF). These mediators stimulate the differentiation of resident fibroblasts and pericytes into myofibroblasts, cells characterized by the expression of alpha-smooth muscle actin (α-SMA) and a heightened capacity for ECM production [78]. Myofibroblasts secrete large quantities of collagen types I and III, leading to tubulointerstitial fibrosis and glomerulosclerosis. The progressive scarring of kidney tissue reduces nephron function, promoting the decline in glomerular filtration rate (GFR) and the development of end-stage renal disease [79].

Oxidative stress exacerbates fibrosis in both the heart and kidneys by perpetuating a pro-inflammatory and profibrotic environment. In brief, ROS, generated by NADPH oxidase, mitochondria, and uncoupled NOS, interact with cellular components to activate fibrogenic pathways [80]. ROS enhance the activity of TGF-β and upregulate pro-inflammatory transcription factors such NF-κB. In endothelial cells, oxidative stress contributes to endothelial-to-mesenchymal transition (EndMT), a process in which endothelial cells lose their normal phenotype and acquire fibroblast-like properties, further increasing the pool of ECM-producing cells [81].

In addition to fibroblast activation, fibrosis involves the breakdown and remodeling of existing ECM, mediated by matrix metalloproteinases (MMPs). While MMPs are necessary for normal ECM turnover, their dysregulation in CKM promotes aberrant remodeling, with an imbalance between MMPs and their tissue inhibitors (TIMPs) favoring excessive ECM deposition [82].

Metabolic factors also contribute significantly to fibrosis. As reported before, hyperglycemia accelerates the production AGEs that are able to induce fibrosis processes. Dyslipidemia adds to the profibrotic environment, as lipid accumulation in non-adipose tissues, such as the myocardium and kidneys, induces lipotoxicity, cellular stress, and apoptosis; the resultant release of pro-inflammatory cytokines and growth factors promotes fibroblast activation and ECM deposition [83,84].

Notably, in the late stages of CKM, fibrosis becomes self-sustaining; the stiff, scarred tissue not only dysregulate normal organ function but also dysregulate mechanosensitive signaling pathways. In the heart, increased stiffness impairs electrical conduction, leading to arrhythmias, while in the kidneys, fibrotic scarring obliterates capillary networks, worsening hypoxia and ischemic injury [85]. This perpetuates a vicious cycle of progressive fibrosis and organ failure, highlighting the importance of early intervention to halt the fibrotic process. Therefore, we can summarize that fibrosis and structural damage in CKM syndrome are the result of chronic pathological stimuli that activate fibroblasts, promote ECM deposition, and change normal tissue architecture [86]. These processes, driven by TGF-β, oxidative stress, and metabolic factors, culminate in irreversible organ dysfunction, underscoring the need for therapeutic strategies to target fibrosis at its molecular origins.

### 3.6. Gut–Kidney–Heart Axis

Interestingly, in CKM, the Gut–Kidney–Heart Axis and microbiome dysbiosis plays a significant role, promoting oxidative stress and inflammation [87]. Dysbiosis, characterized by an imbalance in the composition of gut microbiota, leads to the overproduction of harmful metabolites such as uremic toxins, including indoxyl sulfate and p-cresyl sulfate. These metabolites, once absorbed into the bloodstream, induce oxidative stress in both the cardiovascular and renal systems, exacerbating tissue damage and functional decline [88].

The gut microbiota produces metabolites through the fermentation of dietary fibers, proteins, and other nutrients. However, when dysbiosis occurs—due to factors like a high-fat diet, antibiotic use, or chronic disease states—the microbiota shifts toward an imbalance favoring harmful bacteria and reducing beneficial species. This imbalance results in an increase in the production of toxic compounds, particularly uremic toxins. Uremic toxins, such as indoxyl sulfate and p-cresyl sulfate, are absorbed by the gut and transported to the liver, where they are typically conjugated and excreted [89]. However, in cases of impaired renal function, as seen in CKM syndrome, the kidneys are unable to efficiently eliminate these toxins, leading to their accumulation in the bloodstream. These toxins then act systemically to dysregulate normal cellular processes and increase oxidative stress [90].

Indoxyl sulfate and p-cresyl sulfate exacerbate oxidative stress by promoting the generation of ROS in heart and kidneys; these toxins reduce mitochondrial function, increasing the leakage of electrons during oxidative phosphorylation and enhancing the production of superoxide anions [91]. Excessive ROS production damages cellular components such as lipids, proteins, and DNA, leading to cellular dysfunction, apoptosis, and fibrotic changes [92].

In the cardiovascular system, the accumulation of uremic toxins leads to endothelial dysfunction by reducing NO bioavailability. Notably, NO, which acts as a potent vasodilator and anti-inflammatory molecule, is reduced when superoxide reacts with it to form peroxynitrite. This imbalance promotes vasoconstriction, increased vascular stiffness, and heightened risk of atherosclerosis. Furthermore, the direct toxic effects of uremic toxins on cardiac myocytes contribute to myocardial dysfunction, arrhythmias and heart failure [93].

In the kidneys, uremic toxins exacerbate tubular injury, glomerular sclerosis and interstitial fibrosis. By inducing oxidative stress, these toxins amplify the inflammatory response and accelerate tissue scarring, ultimately leading to a decline in renal function. The interplay between oxidative stress and inflammation worsens the overall condition, making it difficult to restore renal function in CKM syndrome [94].

Moreover, the gut microbiota influences the immune response through the modulation of Toll-like receptors (TLRs) and other pattern recognition receptors (PRRs); notably, dysbiosis contributes to the abnormal activation of these receptors, which initiates inflammatory cascades [95]. TLRs, for example, recognize pathogen-associated molecular patterns (PAMPs) and DAMPs, leading to the release of TNF-α, IL-1, and IL-6. These cytokines further perpetuate oxidative stress, exacerbating the detrimental effects on both cardiovascular and renal tissues [96]. Therefore, microbiome dysbiosis in the context of the Gut–Kidney–Heart Axis leads to the overproduction of uremic toxins, which promote oxidative stress and inflammation. These processes contribute to endothelial dysfunction, fibrotic remodeling, and reduced organ function in the heart and kidneys, highlighting the critical role of gut health in maintaining systemic metabolic and cardiovascular health.

## 4. Common Molecular Pathways Involved in CKM Syndrome and Cancer

CKM syndrome is a complex condition influenced by multiple biochemical pathways that also contribute to CVD and cancer [97]. The dysregulation of these pathways leads to endothelial dysfunction, metabolic disturbances, chronic inflammation, and oxidative stress, which are key drivers of CKM pathology [98]. Understanding the molecular mechanisms of these pathways provides insights into the interplay between CKM syndrome, CVD, and cancer (Table 4). As specified in Section 3.1, RAAS plays a critical role in maintaining blood pressure and fluid balance. Dysregulated RAAS contributes to hypertension, heart failure, and atherosclerosis in CVD, and is implicated in tumor angiogenesis and cancer cell proliferation [99]. Angiotensin II promotes fibrosis, oxidative stress, and inflammation, exacerbating endothelial dysfunction in CKM syndrome; however, aldosterone, ACE, and renin further drive metabolic disturbances and fibrosis, creating a pro-inflammatory and pro-tumorigenic microenvironment.

Moreover, insulin resistance is a hallmark of metabolic syndrome, cancer, and CKM-related complications; dysregulation of insulin signaling contributes to diabetic cardiomyopathy, atherosclerosis, and hyperinsulinemia-driven tumorigenesis, particularly in colorectal and breast cancers [100]. In more detail, the IRS-1/2-PI3K-AKT-mTOR axis is central to insulin-mediated glucose uptake and cellular metabolism. Impaired GLUT4 translocation leads to hyperglycemia, while overactive mTOR signaling enhances tumor growth and metabolic dysfunction, linking CKM syndrome with oncogenic risks. Moreover, chronic inflammation is a shared feature of CVD, CKM syndrome and cancer. NF-κB activation leads to the production of pro-inflammatory cytokines such as IL-6, TNF-α, and IL-1β, which drive atherosclerosis, myocardial fibrosis, and immune evasion in tumors [101]. High COX-2 levels further amplify inflammation and oxidative stress, contributing to endothelial dysfunction and tumor progression. The systemic inflammatory response in CKM patients accelerates cardiovascular damage and cancer-related complications (Table 4).

Another molecular pathway of key importance in CKM is oxidative stress; oxidative stress results from an imbalance between reactive oxygen species (ROS) production and antioxidant defense mechanisms [102].

In CKM syndrome and CVD, excessive ROS from NADPH oxidase (NOX) promotes endothelial dysfunction, mitochondrial damage, and atherosclerosis. Cancer cells exploit oxidative stress to drive DNA mutations and tumor progression; in brief, the Nrf2-Keap1 pathway regulates cellular antioxidant responses, but dysregulation leads to metabolic inflexibility and apoptotic resistance, exacerbating CKM-related metabolic dysfunction [103]. Moreover, altered lipid metabolism is a key contributor to CVD and cancer. Dyslipidemia and increased lipid oxidation promote atherosclerosis and cardiovascular complications in CKM patients. In cancer, lipids serve as an energy source for tumor proliferation, particularly in prostate and breast cancers. PPAR-γ, SREBP-1, and LXR regulate lipid homeostasis, but their dysregulation in CKM syndrome contributes to metabolic disturbances, chronic inflammation, and oncogenesis through abnormal lipid storage and utilization [104].

AMPK acts as an energy sensor, promoting catabolic processes to maintain cellular energy homeostasis. Dysregulated AMPK activity in CKM syndrome leads to metabolic inflexibility and cardiovascular stress. In contrast, mTOR hyperactivation supports cancer cell survival, metabolic adaptation, and proliferation. The ULK1-TSC2-Raptor axis modulates autophagy and cellular growth and its imbalance exacerbates CKM-related complications, linking metabolic syndrome with cancer progression (Table 4). Moreover, TGF-β signaling plays a pivotal role in fibrosis, which is a common feature of CKM syndrome. It contributes to cardiac hypertrophy, kidney fibrosis, and vascular remodeling in CVD. In cancer, TGF-β promotes epithelial–mesenchymal transition (EMT), enhancing metastasis and tumor progression. SMAD2/3 activation drives fibrotic responses, while SMAD7 inhibition exacerbates tissue damage, linking CKM-related organ fibrosis to increased cancer risk [105].

In more detail, HIF-1α is crucial for cellular adaptation to hypoxia; in CVD, it contributes to vascular dysfunction and ischemic injury. In cancer, HIF-1α drives angiogenesis, metabolic reprogramming, and tumor survival (Table 4). The interplay between HIF-1α, VEGF, PHD2, and VHL modulates cellular responses to low oxygen levels. Hypoxia in CKM syndrome exacerbates metabolic imbalances, promoting cardiovascular dysfunction and cancer cell adaptation [106].

As reported in Section 3.6, the gut microbiota significantly influences metabolic and inflammatory pathways. In CKM syndrome, dysbiosis leads to high trimethylamine N-oxide (TMAO) levels, which are associated with atherosclerosis and hypertension. Interestingly, in cancer, microbial dysregulation promotes chronic inflammation and tumor progression; TMAO, FMO3, lipopolysaccharides (LPS), and short-chain fatty acids (SCFA) influence immune responses, linking gut microbiota alterations to CKM-related complications and oncogenic risks [107]. Moreover, chronic kidney dysfunction in CKM syndrome leads to the accumulation of indoxyl sulfate and p-cresol that increases vascular calcification, oxidative stress, and chronic inflammation, exacerbating cardiovascular risk. In cancer, they promote chronic inflammation and endothelial dysfunction, facilitating tumor progression [108]. Dysregulation of Klotho and eNOS further impairs endothelial function, linking CKM syndrome to heightened oncogenic potential.

## 5. Anticancer Therapies and Risk of CKM Syndrome

**Figure 2:**
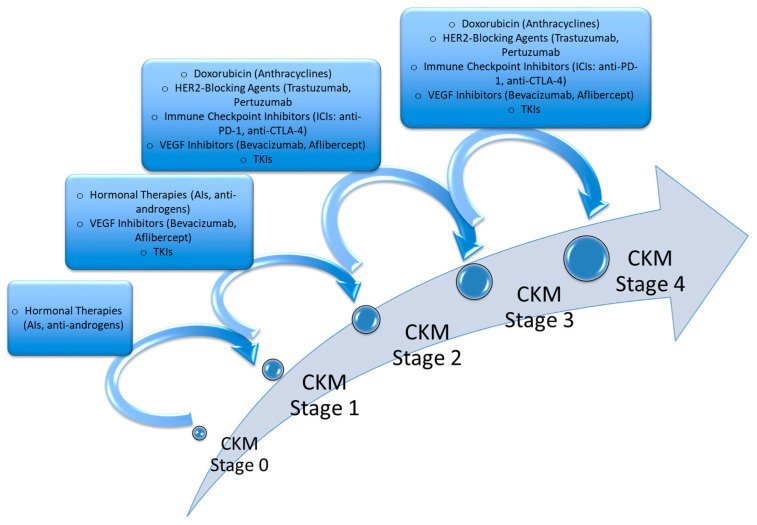
Schematic description of CKM progression in cancer patients (from stage 0 to stage 4) and impact of anticancer therapies.

Notably, CKM syndrome plays a significant role in cardio-oncology, where the interaction of cardiovascular health and cancer treatment has become increasingly critical (Figure 2). Anticancer therapies, including chemotherapy, radiation, and targeted therapies, often exacerbate pre-existing cardiovascular and kidney diseases or induce new complications, making the management of CKM essential for optimizing outcomes in cancer patients [109] (Table 5).

Chemotherapy can lead to many cardiorenal complications, including cardiomyopathy, heart failure, arrhythmias, and renal impairment; these effects are exacerbated in patients already at risk due to pre-existing CKM conditions [110]. The chronic inflammation, oxidative stress and metabolic diseases in CKM contribute to the susceptibility of cancer patients to CTRCD. Anthracyclines generate ROS, lipid peroxidation, and high levels of NLRP-3 and MyD-88 that damage mitochondrial function and contribute to cardiac cell apoptosis; this oxidative stress amplifies the already heightened state of systemic inflammation in CKM, where pro-inflammatory cytokines, like TNF-α and IL-6, are upregulated [111]. These cytokines exacerbate endothelial dysfunction, impair NO signaling and promote vascular stiffness, further compromising cardiovascular health.

Similarly, anticancer therapies can lead to chronic kidney disease through direct nephrotoxicity, including direct damages of tubular and glomerular cells. In patients with CKM, the compromised renal function is worsened, as the kidneys struggle to excrete indoxyl sulfate and p-cresyl sulfate, increasing oxidative stress and contributing to kidney fibrosis and sarcopenia [112].

In clinical oncology, the integration of cardio-oncology into the management of cancer patients with CKM syndrome is imperative to mitigate therapy-related cardiovascular and renal complications [113]. A multidisciplinary approach that includes routine surveillance of cardiovascular and renal function, individualized therapeutic strategies and targeted interventions for metabolic dysfunction is critical in reducing the incidence of CTRCD. Optimizing CKM management allows clinicians to attenuate the risk of cardiotoxicity and nephrotoxicity, ultimately improving long-term survival and quality of life in cancer survivors [114].

The cumulative risk of cardiorenal toxicity associated with contemporary oncologic therapies—including HER2-targeted agents, anthracyclines, VEGF inhibitors, cyclin-dependent kinase 6 (CDK6) inhibitors, and hormonal therapies—stems from their capacity to exacerbate preexisting cardiovascular and renal dysfunction [115]. While these agents are integral to oncologic treatment paradigms, their mechanistic interplay with the pathophysiology of CKM syndrome leads to an amplified risk of toxicity. This complex interplay underscores the necessity for vigilant risk stratification and proactive management strategies in this vulnerable patient population.

### 5.1. How Do HER2-Blocking Agents (e.g., Trastuzumab, Pertuzumab) Exacerbate CKM Syndrome?

CKM can contribute to an increased susceptibility to heart failure and arrhythmias in patients receiving HER2 inhibitors [116] (Table 5).

HER2-targeted therapies, such as trastuzumab, reduce myocardial homeostasis by inhibiting HER2-mediated cardioprotective signaling, which is essential for cardiomyocyte survival. This inhibition exacerbates endothelial dysfunction in CKM patients reducing NO bioavailability and promoting vascular inflammation which leads to microvascular ischemia and impaired myocardial perfusion [117] (Table 5). The preexisting oxidative stress and systemic inflammation observed in CKM further amplify trastuzumab-induced mitochondrial dysfunction, increasing ROS production and impairing mitochondrial bioenergetics. This leads to excessive apoptosis of cardiomyocytes and progressive left ventricular dysfunction [118]. Moreover, the neurohormonal dysregulation in CKM intensifies the cardiotoxic effects of HER2 blockade, increasing the risk of ventricular arrhythmias and heart failure. The cumulative impact of these pathophysiological alterations underscores the need for close cardiovascular surveillance and proactive intervention in patients with CKM undergoing HER2-targeted cancer therapy (Table 5).

Epidemiological data indicate a significant prevalence of CKM among cancer patients, which may exacerbate the cardiotoxic effects of HER2-targeted therapies [119]. A recent study reported that 45.44% of cancer patients exhibit metabolic syndrome (MetS), and 19.23% have CVD; these conditions are components of CKM syndrome and are known to increase the risk of cardiotoxicity when patients are treated with HER2 inhibitors [120]. The incidence of cardiotoxicity in patients receiving HER2-targeted therapies varies, with studies reporting rates ranging from 1.7% to higher percentages, depending on patient populations and definitions used. Risk factors such as older age, diabetes mellitus, decreased glomerular filtration rate, and a history of heart disease have been associated with an increased likelihood of developing cardiotoxicity [121].

Given the high prevalence of CKM conditions in the cancer population and their association with increased cardiotoxic risk, it is imperative to implement vigilant cardiovascular monitoring and tailored therapeutic strategies for patients undergoing HER2-targeted therapy.

### 5.2. How Do Anthracyclines (e.g., Doxorubicin, Epirubicin) Exacerbate CKM Syndrome?

Anthracyclines, such as doxorubicin and epirubicin, are effective chemotherapeutic agents but are associated with a risk of cardiotoxicity, which can lead to heart failure [122]. The incidence of anthracycline-induced heart failure is dose-dependent, with reported rates of 7% at a cumulative dose of 150 mg/m^2^, 18% at 350 mg/m^2^, and 65% at 550 mg/m^2^ [123] (Figure 2).

Patients with pre-existing CKM conditions—including hypertension, diabetes mellitus, and obesity—are at an increased risk of developing anthracycline-induced cardiotoxicity. Biochemically, anthracyclines increase NLRP3/IL-1 pathways in myocardial cells that in patients with CKM is common upregulated in both myocardial and renal tissue [124]. A meta-analysis indicated that patients with hypertension have a higher likelihood of experiencing cardiotoxicity following anthracycline therapy.

Similarly, diabetes mellitus and obesity have been identified as significant risk factors for anthracycline-induced cardiotoxicity. The presence of CKM conditions may exacerbate the cardiotoxic effects of anthracyclines through mechanisms such as increased oxidative stress, endothelial dysfunction, and neurohormonal activation [125]. These factors can potentiate myocardial injury, leading to a higher incidence of heart failure in this patient population. Therefore, it is crucial to implement vigilant cardiovascular monitoring and personalized therapeutic strategies for patients with CKM undergoing anthracycline-based chemotherapy to mitigate the heightened risk of cardiac dysfunction [126].

### 5.3. Do VEGF Inhibitors (e.g., Bevacizumab, Aflibercept) Increase the Risk of CKM?

Vascular Endothelial Growth Factor (VEGF) inhibitors, such as bevacizumab and aflibercept, are pivotal in cancer therapy due to their anti-angiogenic properties [127] (Figure 2).

However, their use is associated with cardiovascular toxicities, particularly in patients with pre-existing CKM. VEGF inhibitors inhibit angiogenesis by blocking VEGF signaling pathways, essential for vascular homeostasis [128]. Notably, inhibition of VEGF reduces nitric oxide production and increases endothelin-1 levels, leading to vasoconstriction and high blood pressure. Hypertension is the most frequent cardiotoxicity observed with VEGF signaling pathway (VSP) inhibitors, with a reported incidence ranging from 19% to 47%. Moreover, VEGF inhibition can lead to left ventricular dysfunction and heart failure [129]. The incidence of heart failure in patients receiving VEGF inhibitors varies, with some studies reporting rates up to 3% (Table 5).

Moreover, VEGF inhibitors have been associated with an increased risk of arterial and venous thromboembolic events. A study reported that angiogenesis inhibitors were associated with increased risks of major adverse cardiovascular events, including heart failure, myocardial infarction, stroke, and venous thromboembolism [130]. Notably, patients with CKM are at an increased risk of VEGF inhibitor-induced cardiotoxicity. The presence of these comorbidities exacerbates endothelial dysfunction and heightens susceptibility to adverse cardiovascular events. Therefore, given the prevalence of CKM conditions in the oncology population, it is imperative to implement vigilant cardiovascular monitoring and personalized therapeutic strategies for patients undergoing VEGF inhibitor therapy to mitigate the heightened risk of cardiac dysfunction [131].

### 5.4. Do Hormonal Therapies (e.g., Aromatase Inhibitors, Anti-Androgens) Increase the Risk of CKM Syndrome?

Hormonal therapies, including aromatase inhibitors and anti-androgens, are cornerstone treatments for hormone-sensitive malignancies. However, these therapies can induce metabolic alterations such as increased adiposity, insulin resistance, and nonalcoholic fatty liver disease (NAFLD), which may increase CVD risk, especially in patients with pre-existing CKM conditions [132]. Aromatase inhibitors (AIs) reduce estrogen levels by inhibiting the aromatase enzyme, leading to decreased estrogen-mediated regulation of adipose tissue distribution and insulin sensitivity. This reduction can result in increased central adiposity and insulin resistance. Studies have shown that estrogen deficiency is associated with the development of NAFLD, as estrogens play a protective role against hepatic fat accumulation [133]. Moreover, anti-androgens inhibit androgen receptor signaling, which is crucial for maintaining lean body mass and metabolic homeostasis.

Androgen deficiency or blockade has been linked to increased fat mass, insulin resistance, and the development of NAFLD [134]. Research indicates that androgen dysfunction contributes to hepatic steatosis and metabolic dysregulation. The prevalence of obesity and insulin resistance is notably higher in patients undergoing hormonal therapies. Obesity is a significant risk factor for both CKM and CVD. Insulin resistance exacerbates hepatic gluconeogenesis and lipogenesis, contributing to NAFLD and increasing CVD risk [135] (Figure 2).

Moreover, NAFLD is prevalent among patients receiving hormonal therapies, with studies indicating a higher incidence in this population. NAFLD is an independent risk factor for CVD and is often associated with metabolic syndrome components, including dyslipidemia and hypertension [136]. Patients with pre-existing CKM conditions are particularly susceptible to the compounded metabolic and cardiovascular effects of hormonal therapies. The additive impact of therapy-induced metabolic disturbances on existing CKM pathology can lead to a heightened risk of adverse cardiovascular events [137].

Therefore, it is imperative to implement comprehensive monitoring and management strategies, including regular assessment of metabolic parameters, lifestyle interventions to mitigate weight gain and insulin resistance, and consideration of alternative therapeutic regimens when appropriate. In summary, while hormonal therapies are essential in managing hormone-sensitive cancers, their potential to induce metabolic dysregulation necessitates careful consideration, especially in patients with CKM conditions [138]. A multidisciplinary approach is crucial to balance oncologic efficacy with the minimization of cardiovascular risk (Table 5).

### 5.5. Does Immunotherapy (ICIs) Pose Additional Cardiovascular Risks for CKM Syndrome Patients?

Immune checkpoint inhibitors (ICIs) have revolutionized cancer treatment, particularly in melanoma, lung cancer, and bladder cancer [139]. However, their use is associated with immune-related adverse events (IRAEs), which can affect various organ systems, including cardiovascular and renal functions. When combined with CKM syndrome, the additive risk of cardiorenal toxicity becomes significant due to overlapping pathways of inflammation, immune activation and metabolic dysfunction. In brief, anti-PD-1, anti-PD-L1, and anti-CTLA-4 antibodies target immune checkpoint pathways to enhance the body’s immune response against cancer cells. While effective in treating cancers, these therapies can trigger immune dysregulation, leading to systemic inflammation and increased oxidative stress [140].

In CKM patients, where chronic inflammation, endothelial dysfunction, and metabolic diseases are already present, ICIs can exacerbate these processes, further promoting cardiovascular and renal damages. The mechanisms of cardiorenal toxicity with ICIs involve inflammatory cytokine release, endothelial dysfunction, oxidative stress renal dysfunction, electrolyte imbalance, and fluid retention [141] (Table 5).

In more detail, ICIs enhance immune activation by blocking immune checkpoint pathways, which can lead to the release of pro-inflammatory cytokines like IL-1β, IL-6, TNF-α, and interferon-gamma (IFN-γ). In CKM patients, whose inflammatory state is very high, the additional release of these cytokines contributes to a heightened state of oxidative stress and tissue damage, particularly in cardiovascular and renal tissues [142]. Moreover, ICIs can exacerbate endothelial damage by promoting immune cell infiltration and the formation of pro-inflammatory mediators. The already-compromised vascular health in CKM patients worsens with further endothelial injury, increasing the risk of atherosclerosis, thromboembolic events, and impaired renal perfusion [143]. Moreover, ICIs stimulate immune responses that increase ROS production, which can overwhelm the antioxidant defense system. In CKM, where metabolic abnormalities and mitochondrial dysfunction contribute to ROS generation, the combination of these therapies amplifies oxidative stress, leading to cellular damage and fibrosis in both cardiac and renal tissues [144].

Moreover, ICIs can lead to acute kidney injury (AKI) through immune-mediated mechanisms such as T cell infiltration, tubular injury, and glomerular inflammation. In patients with CKM, whose kidneys are already under strain, the additional renal injury accelerates the decline in kidney function, contributing to worsening CKM progression [145]. Moreover, ICIs can cause electrolyte disturbances, including hypophosphatemia, hyponatremia and hyperkalemia, which are exacerbated in patients with pre-existing CKM; these effects can further strain cardiovascular and renal systems, leading to fluid overload and worsening heart and kidney function [146] (Table 5)

The use of ICIs in monotherapy regimen or combined to HER2-blocking agents, anthracyclines, VEGF inhibitors, CDK6 inhibitors, and hormonal therapies creates a synergistic risk for cardiorenal toxicity in cancer patients. Each therapy independently impacts vascular function, oxidative stress, inflammation, and metabolic homeostasis, and their combination amplifies these effects. The additive burden increases the risk of heart failure, arrhythmias, and hypertension, associated with renal dysfunction, leading to an increased likelihood of end-stage organ failure in CKM patients (Table 5).

Cancer therapies, including hormonal therapies, doxorubicin, HER2-blocking agents, immune checkpoint inhibitors (ICIs), and VEGF inhibitors (VEGFi), can accelerate the progression of CKM syndrome by worsening metabolic, renal, and cardiovascular dysfunction. Below is an overview of how each therapy influences the transition between CKM stages.

As illustrated in Table 5, hormonal therapies primarily influence early-stage CKM (0 → 2) by worsening metabolic dysfunction; doxorubicin and HER2-blocking agents could push patients from Stage 2 to Stage 4 due to direct cardiotoxic effects on cardiomyocytes. Moreover, VEGF inhibitors affect all CKM stages by causing hypertension, endothelial dysfunction, and renal injury, due to the high expression of VEGFR in endothelial cells in myocardium. Instead, ICIs could drive inflammation and cardiorenal toxicity, accelerating the transition to later stages (2 → 4) due to endocrine and cardiorenal toxicities. This highlights the need for early metabolic screening, cardio-oncology monitoring, and personalized CKM management to mitigate treatment-induced toxicity in cancer patients.

## 6. Pharmacological and Non-Pharmacological Therapies for CKM in Cancer Patients

In brief, CKM syndrome in cancer patients requires a comprehensive approach involving both pharmacological and non-pharmacological therapies. Pharmacological management includes new antidiabetic agents like SGLT2 inhibitors, which offer both cardioprotective and nephroprotective benefits, PCSK9i, GLP1-RA, and others (Table 6). Non-pharmacological strategies focus on dietary modifications, weight management, regular physical activity, and smoking cessation. Multidisciplinary care, integrating oncologists, nephrologists, and cardiologists, is essential for optimizing outcomes in CKM syndrome among cancer patients (Table 6).

### 6.1. Sodium-Glucose Co-Transporter 2 Inhibitors (SGLT2i)

Sodium-glucose co-transporter 2 inhibitors (SGLT2i) offer a promising therapeutic approach for patients with CKM syndrome, especially when integrated into cancer care [147]. The SGLT2i have been shown to provide multiple benefits in managing cardiorenal complications through their pleiotropic biochemical mechanisms. First, by inhibiting SGLT2 in the proximal renal tubules, these drugs promote the excretion of glucose through the urine. This lowers blood glucose levels and alleviates the associated insulin resistance that is prevalent in CKM [148]. In cancer patients, where metabolic dysregulation and hyperglycemia are common complications, SGLT2 inhibitors help mitigate the metabolic disturbances caused by hyperinsulinemia and the associated inflammatory state [149] (Table 6).

Insulin resistance is a significant driver of oxidative stress and inflammation in CKM patients. High insulin levels contribute to the overactivation of pathways such as the protein kinase B (Akt) and mammalian target of rapamycin (mTOR) signaling pathways, which promote cellular proliferation and survival. In the context of cancer, where cell proliferation is a key component of disease progression, this metabolic dysfunction can exacerbate the tumor microenvironment and fuel cancer progression [150]. By reducing insulin levels, SGLT2 inhibitors help to restore metabolic balance and reduce this pathway’s contribution to cancer progression.

SGLT2i have been shown to reduce oxidative stress, a critical factor contributing to cardiovascular and renal dysfunction in CKM patients. Chronic oxidative stress, driven by mitochondrial dysfunction and the production of ROS, accelerates cell damage and promotes inflammatory responses. This imbalance is particularly pronounced in cancer patients undergoing aggressive therapies, such as chemotherapy or immune checkpoint inhibitors, which further induce mitochondrial stress and ROS production [151].

By decreasing the reabsorption of glucose, SGLT2 inhibitors help to lower intracellular glucose and lipid levels, reducing the metabolic precursors for ROS generation. Additionally, SGLT2i activate antioxidant pathways, such as the activation of the nuclear factor erythroid 2-related factor 2 (Nrf2) pathway, which enhances the expression of antioxidant enzymes like superoxide dismutase (SOD) and catalase [152]. These enzymes mitigate the harmful effects of ROS, protecting both cardiovascular and renal tissues from oxidative damage and improving overall organ function.

In patients with CKM, SGLT2i provide substantial cardiovascular protection through their ability to reduce cardiovascular mortality and morbidity. The biochemical basis for this benefit stems from their effects on several pathways associated with cardiovascular health. First, SGLT2i reduce the volume of circulating blood and lower blood pressure through osmotic diuresis and natriuresis, which helps alleviate the strain on the cardiovascular system [153]. This is particularly valuable in cancer patients, many of whom are at risk for fluid retention and volume overload due to both cancer therapies and underlying metabolic imbalances.

Furthermore, SGLT2i improve endothelial function by promoting NO bioavailability. Nitric oxide is a crucial molecule for vasodilation and maintaining vascular integrity and its preservation is essential for reducing the risk of atherosclerosis and arterial stiffness [154]. In CKM patients, where endothelial dysfunction is a hallmark feature, this benefit is amplified. Enhanced vascular health protects against cardiovascular events such as heart attacks, arrhythmias, and heart failure. Moreover, SGLT2i exerts renal protection in direct and indirect manner; in fact they are able to reduce intraglomerular pressure and tubule-interstitial damages, which are critical contributors to progressive kidney disease [155]. Anticancer therapies, particularly those with nephrotoxic potential such as platinum-based agents and ICIs, frequently impair kidney function, leading to acute kidney injury (AKI) and CKD (Table 6).

The reduction in oxidative stress and inflammation through SGLT2 inhibition directly reduces kidney fibrosis; additionally, SGLT2i are associated with improvements in albuminuria, providing further protection against CKD progression in patients with cancer [156]. Chronic inflammation is a central feature of CKM syndrome, driven by metabolic dysfunction and immune dysregulation. In cancer patients, where therapies further exacerbate inflammatory responses, the use of SGLT2i provides an anti-inflammatory benefit. By reducing hyperglycemia and suppressing the activation of inflammatory pathways, such as NF-κB and IL-6, SGLT2i reduce systemic inflammation [157].

Moreover, during ICIs, which often lead to immune dysregulation and autoimmune-like responses, SGLT2i could be able to reduce IRAEs, including myocarditis, nephritis and other organ-specific inflammation, thereby improving the safety and efficacy of cancer therapies. In summary, SGLT2i offer multifaceted benefits for patients with CKM and cancer by addressing metabolic dysfunction, oxidative stress, cardiovascular strain, and renal impairment [158]. Through these mechanisms, SGLT2i provide a valuable tool for managing the complex challenges of treating cancer in patients with underlying CKM syndrome, ultimately improving both QoL and anticancer outcomes.

### 6.2. Proprotein Convertase Subtilisin/Kexin Type 9 Inhibitors (PCSK9i)

Proprotein convertase subtilisin/kexin type 9 inhibitors (PCSK9i) are a class of monoclonal antibodies (e.g., alirocumab, evolocumab) or small interfering RNA (siRNA) agents (e.g., inclisiran) that reduce LDL-C levels by preventing PCSK9-mediated degradation of LDL receptors [159]. These drugs have demonstrated significant cardiovascular benefits by lowering LDL-C and reducing atherosclerotic cardiovascular disease (ASCVD) risk. CKM syndrome involves CVD, CKD, and metabolic disorders (e.g., type 2 diabetes, obesity, dyslipidemia) that collectively accelerate atherosclerosis and increase cardiovascular mortality (Table 6).

Patients with CKM are often at high or very high ASCVD risk, necessitating aggressive lipid-lowering therapy [121]. PCSK9i offer an effective and well-tolerated option, especially in patients who have statin intolerance, require additional LDL-C lowering or have severe CKD where statins may have limited benefits. The role of PCSK9i in this population requires careful consideration of several factors. Cancer patients with CKM syndrome are already predisposed to cardiovascular events, making lipid management very crucial. PCSK9i effectively lower LDL-C, reducing the risk of chemotherapy-induced vascular events such as myocardial infarction or stroke [160]. They have shown benefits in reducing major adverse cardiovascular events (MACE), which is particularly relevant for cancer survivors at high risk of CVD. PCSK9 plays a role in antigen presentation and immune regulation [161].

There is ongoing investigation into whether PCSK9 inhibition affects immune responses in cancer patients receiving immunotherapy. Some preclinical studies have suggested a potential link between PCSK9 and tumor growth through its effects on LDL receptor-mediated cholesterol homeostasis [162]. However, large clinical trials have not demonstrated increased cancer incidence with PCSK9i use.

PCSK9 inhibitors are generally well tolerated, with low rates of myalgia, hepatotoxicity, or renal dysfunction, making them a safer alternative to statins in patients with CKD or multiple comorbidities.

Cancer treatments often induce metabolic alterations, including dyslipidemia; in fact, hormonal therapies (e.g., androgen deprivation therapy in prostate cancer, aromatase inhibitors in breast cancer) can worsen lipid profiles [163]. Moreover, corticosteroids used in chemotherapy regimens increase triglycerides and LDL-C. The PCSK9i provide a non-statin alternative for lipid control without significant drug–drug interactions with chemotherapy agents. Notably, PCSK9i should be considered in cancer patients with CKM syndrome who have high LDL-C despite maximally tolerated statins or in those who are statin-intolerant. However, more studies are required to determine the long-term impact of PCSK9 inhibition on cancer progression and outcomes in patients undergoing active cancer treatment [164] (Table 6).

We assess that PCSK9 i represent a promising lipid-lowering strategy in patients with CKM syndrome and cancer; they provide cardiovascular protection, have no renal toxicity, and are generally well tolerated. While safety concerns regarding immune modulation and cancer progression exist, current evidence supports their use in high-risk cancer patients with CKM syndrome [164]. Further clinical research will be essential to optimize their role in this complex patient population.

### 6.3. Soluble Guanylate Cyclase Activators

Soluble guanylate cyclase activators (sGCa) represent a promising therapeutic strategy for managing cardiovascular and renal dysfunction in patients with CKM syndrome, particularly when combined with cancer care [165]. In brief, sGC activators work by directly stimulating soluble guanylate cyclase, an essential enzyme responsible for the conversion of guanosine triphosphate (GTP) into cyclic guanosine monophosphate (cGMP). cGMP acts as a crucial signaling molecule that regulates various physiological processes, including vasodilation, anti-inflammatory responses, and mitochondrial function [166]. In patients with CKM, the endothelial dysfunction and impaired NO signaling present significant challenges to maintaining vascular health, which is further exacerbated by the use of cancer therapies that can promote oxidative stress and vascular injury (Table 6).

Anticancer therapies, particularly those that involve high-dose chemotherapy or ICIs, can lead to a decrease in endothelial NO production due to increased oxidative stress and inflammation. In patients with pre-existing CKM, where vascular health is already compromised, the additional decline in NO signaling amplifies the risk of cardiovascular events such as hypertension, atherosclerosis, and heart failure [167]. sGC activators restore and enhance NO signaling, promoting vasodilation, reducing vascular stiffness, and protecting against cardiovascular complications. This improvement in NO signaling is critical in patients undergoing cancer treatment, where cardiovascular strain is often exacerbated by fluid retention, hypertension and myocardial damage [168].

Chronic oxidative stress and inflammation are central to the pathogenesis of CKM, and these processes are further intensified in cancer patients undergoing aggressive therapies; sGC activators counteract oxidative stress by promoting the production of cGMP, which, in turn, activates the cGMP-dependent protein kinase (PKG). PKG modulates antioxidant pathways and enhances cellular defenses against ROS-mediated damage [169]. By reducing the accumulation of ROS, sGC activators protect cellular structures such as lipids, proteins, and DNA, which are critical for maintaining the integrity of cardiovascular and renal tissues.

In addition to mitigating oxidative stress, sGC activators modulate inflammatory responses by suppressing the expression of pro-inflammatory cytokines like CCL-2, IL-1α, IL-1β, IL-6, IL-12, IL17-α, TNF-α, and G-CSF. These cytokines are very high in plasma of patients with CKM; therefore, through the reduction of inflammatory signaling, sGC activators help to prevent the downstream effects of chronic inflammation, such as endothelial damage, fibrosis, and organ dysfunction, which are particularly concerning in cancer patients with pre-existing cardiovascular or renal conditions [170].

Moreover, sGC activators are particularly beneficial for cardiovascular health in patients with CKM by improving heart function and reducing strain on the cardiovascular system. Through the enhancement of cGMP signaling, sGC activators promote vasodilation and reduce systemic vascular resistance, leading to decreased cardiac workload and lower blood pressure. This is essential in cancer patients who may be at risk for chemotherapy-induced cardiotoxicity or cardiovascular strain from immune-related toxicities [171].

Furthermore, sGC activators support myocardial function by improving mitochondrial efficiency and reducing myocardial hypertrophy—a condition commonly seen in CKM patients with underlying metabolic disturbances. Through their protective effects on mitochondrial integrity and energy production, sGC activators prevent the loss of cardiac function associated with oxidative stress and inflammation. This is especially important for cancer patients receiving therapies known to impair cardiovascular health, such as anthracyclines or HER2-targeted therapies, which can exacerbate myocardial dysfunction [172].

In more detail, sGC activators also offer significant benefits in protecting renal function in CKM patients undergoing cancer treatments. sGC activators work by improving renal perfusion and reducing tubulointerstitial fibrosis, which are critical pathways involved in kidney dysfunction. In brief, cGMP signaling enhances renal function by promoting vasodilation within the glomeruli and reducing inflammation in the renal parenchyma [173]. By mitigating oxidative stress and inflammation in the renal tissue, sGC activators prevent the progression of CKD, which is a common complication in patients with CKM. Additionally, the activation of sGC preserves the filtration capacity of the kidneys, helping to maintain metabolic homeostasis and electrolyte balance, which are often dysregulated in patients receiving nephrotoxic cancer therapies.

sGC activators offer a comprehensive approach to managing the multi-faceted challenges faced by patients with CKM undergoing anticancer treatment. By addressing both cardiovascular and renal health, sGC activators play a critical role in improving the overall clinical outcomes of cancer patients, reducing the risk of adverse events associated with cancer therapies, and enhancing QoL [174]. In conclusion, sGCa could provide a powerful tool in the management of CKM syndrome in cancer patients. Through their biochemical modulation of NO signaling, reduction of oxidative stress, and regulation of inflammation, sGC activators enhance cardiovascular and renal function, ultimately improving patient outcomes in the context of complex and multi-systemic cancer care.

### 6.4. Glucagon-like Peptide-1 Receptor Agonists

Glucagon-like peptide-1 receptor agonists (GLP-1RAs) have emerged as a valuable therapeutic option for managing CKM syndrome, particularly during anticancer therapies. GLP-1RAs offer a unique biochemical approach to mitigating these complications by addressing multiple aspects of CKM, including metabolic control, inflammation and organ protection [175] (Table 6). In brief, GLP-1RAs exert their therapeutic effects by enhancing glucose-dependent insulin secretion, suppressing glucagon secretion and slowing gastric emptying. This hormonal action helps to maintain glucose homeostasis and reduce hyperglycemia, a key driver of metabolic complications. In cancer patients, where therapies can impair insulin sensitivity or induce adverse metabolic side effects, maintaining optimal glucose regulation becomes crucial in preventing further cardiovascular and renal complications [176]. Additionally, GLP-1RAs promote weight loss and fat mass reduction, which is particularly beneficial for cancer patients with metabolic abnormalities associated with obesity or cachexia. Excessive adiposity can contribute to systemic inflammation, oxidative stress, and vascular dysfunction, all of which are amplified in CKM patients undergoing cancer treatment. By addressing these metabolic disturbances, GLP-1RAs improve overall metabolic health, thereby reducing the strain on cardiovascular and renal systems [177]. As reported before, chronic inflammation and oxidative stress are central contributors to the pathophysiology of CKM syndrome, and these processes are further intensified in cancer patients during chemo-radiotherapy regimens. GLP-1RAs exerts anti-inflammatory effects by modulating the expression of pro-inflammatory cytokines that play a key role in vascular damage, fibrosis, and organ dysfunction. Furthermore, GLP-1RAs enhance the expression of antioxidant enzymes, including SOD and glutathione peroxidase, which combat the harmful effects of ROS. In CKM patients, where oxidative stress plays a critical role in the progression of cardiovascular and renal disease, reducing ROS accumulation through GLP-1RAs helps protect against cellular damage and organ dysfunction [178]. Moreover, GLP-1RAs promote vasodilation via the release of NO from endothelial cells, reducing systemic vascular resistance, and improving blood flow. This effect is crucial for managing hypertension, a common complication in CKM patients undergoing cancer treatment, where fluid retention and impaired vascular function can strain the cardiovascular system [179].

In addition to enhancing vascular function, GLP-1RAs reduce the risk of atherosclerosis and myocardial damage by reducing circulating levels of LDL-C and triglycerides, while increasing HDL-C. These lipid-modulating effects are particularly beneficial in cancer patients receiving anticancer therapies that can exacerbate lipid abnormalities and cardiovascular stress. By stabilizing lipid profiles, GLP-1RAs protect against the development of atherosclerosis and cardiovascular events, such as heart attacks and stroke [180].

Moreover, GLP-1RAs have been shown to exert protective effects on the kidneys by reducing glomerular hyperfiltration and lowering intraglomerular pressure. In addition to lowering the burden of hyperglycemia, GLP-1RAs reduce proteinuria and slow the progression of kidney damage through their anti-inflammatory and anti-fibrotic properties [181]. They modulate various cellular pathways involved in renal inflammation and fibrosis, including TGF-β signaling pathway, which plays a key role in kidney scarring. By preventing the progression of kidney disease, GLP-1RAs contribute to maintaining renal function in cancer patients, minimizing the risk of end-stage renal disease (ESRD).

Like SGLT2i, GLP-1RAs offer a complementary approach by providing metabolic control, anti-inflammatory effects and organ protection without interfering with the efficacy of cancer treatments. Unlike some targeted therapies or chemotherapy regimens that exacerbate CKM-related complications, GLP-1RAs work synergistically to enhance the overall health of cardiovascular and renal systems [182,183]. Moreover, GLP-1RAs have shown promise in improving patient outcomes by reducing the incidence of MACE and preserving renal function. By integrating GLP-1RAs into the management of CKM and cancer, clinicians could optimize the therapeutic balance between effective cancer treatment and the mitigation of CKM-related risks (Table 6).

In summary, GLP-1 receptor agonists offer a multifaceted biochemical approach to managing cardiovascular and renal complications in patients with CKM and cancer. Through their regulation of glucose metabolism, reduction of oxidative stress and promotion of anti-inflammatory and vascular-protective pathways, GLP-1RAs contribute significantly to preserving cardiovascular and renal function, ultimately enhancing the overall well-being of cancer patients facing the challenges of complex metabolic and organ dysfunction.

### 6.5. Anti-Inflammatory Diet

A diet at low glycemic index (GI) can be highly beneficial for patients with CKM syndrome, particularly those undergoing anticancer treatment. A low glycemic index diet offers a biochemical approach to managing these complications by promoting better metabolic control, reducing inflammation and protecting organ health [184] (Table 6).

One of the primary mechanisms through which a low glycemic index diet benefits CKM patients is through its ability to regulate blood glucose levels and insulin sensitivity. Glycemic index refers to how quickly carbohydrates in food are broken down into glucose and absorbed into the bloodstream. Foods with a low glycemic index are digested more slowly, leading to a more gradual and sustained increase in blood glucose levels, which helps maintain stable insulin levels [185].

As reported before, in patients with CKM, hyperglycemia and insulin resistance are significant contributors to metabolic dysfunction. High AGEs can promote the activation of various inflammatory and oxidative stress pathways, worsening cardiovascular and renal health. Insulin resistance further exacerbates these effects by triggering the overproduction of insulin, which drives inflammatory cytokines and impairs endothelial function, contributing to vascular damage [186]. A low glycemic index diet reduces these fluctuations in blood sugar and insulin levels, minimizing the biochemical triggers of inflammation and oxidative stress. This stable metabolic state helps preserve the integrity of cardiovascular and renal tissues, mitigating the progression of CKM-related complications [187].

Moreover, a low glycemic index diet is able to reduce NAFLD, visceral obesity, sarcopenic obesity, and HOMA score. Additionally, the slow absorption of carbohydrates from low glycemic index foods limits the rapid generation of glucose, reducing the oxidative stress burden on cells [188]. This is particularly important in CKM patients, where oxidative stress drives damage to cellular structures, including lipids, proteins, and DNA, accelerating the decline of cardiovascular and renal function. By reducing AGEs accumulation, a low glycemic index diet preserves mitochondrial function and minimizes cellular damage, protecting against long-term complications in both cancer and CKM patients.

From a biochemical point of view, an anti-inflammatory and low GI diet can play a crucial role in mitigating the cardiotoxicity induced by anticancer drugs in patients with CKM syndrome [189]. An anti-inflammatory diet rich in omega-3 fatty acids (e.g., from fish and flaxseeds) and polyphenolic compounds (e.g., from fruits, vegetables, and whole grains) can suppress the expression of pro-inflammatory cytokines and inflammatory mediators. Omega-3 fatty acids, in particular, reduce the activity of NF-κB, a key transcription factor involved in the inflammatory response, thereby reducing the release of inflammatory cytokines and oxidative stress. Furthermore, polyphenols (like resveratrol and flavonoids) act as antioxidants and have been shown to decrease oxidative damage and improve mitochondrial function [190].

Moreover, high GI foods like white rice cause rapid increases in blood glucose and insulin levels, leading to hyperinsulinemia, which not only worsens insulin resistance but also promotes the activation of pathways like mTOR and PKC, both of which are involved in pathogenesis of CTRCD. Instead, low-GI foods, especially whole grains, legumes and non-starchy vegetables, ensure more gradual increases in blood glucose, insulin, IGF-1, and leptin levels [191]. This dietary pattern reduces hyperinsulinemia and improves insulin sensitivity, which has been linked to a reduction in the activation of the aforementioned detrimental pathways. Lowering insulin and leptin levels through a low-GI diet also decreases the overall workload on the heart, which is crucial in a setting where anticancer drugs may induce cardiomyocyte damage through mitochondrial dysfunction and excessive ROS production.

Moreover, a diet rich in antioxidants (e.g., vitamins C and E, selenium, and flavonoids) reduces MDA, 4-HNA generation. These antioxidants scavenge free radicals, preventing oxidative damage to the myocardium and improving mitochondrial function. Furthermore, omega-3 fatty acids, through their action on peroxisome proliferator-activated receptors (PPARs), can enhance the expression of genes involved in oxidative stress defense and mitochondrial biogenesis [192]. As a result, the cardioprotective effects of the anti-inflammatory diet help mitigate the adverse impact of ROS induced by anticancer therapies. An anti-inflammatory, low-GI diet enhances endothelial nitric oxide synthase (eNOS) activity, which is crucial for maintaining vascular health. Omega-3 fatty acids, polyphenols, and poly-unsaturated fatty acids have been shown to upregulate eNOS expression, resulting in improved NO production and vasodilation [193]. These dietary components also decrease the expression of adhesion molecules such as VCAM-1 and ICAM-1, which are implicated in leukocyte adhesion and endothelial injury (Table 6).

Notably, a low-GI diet can activate the AMPK (AMP-activated protein kinase) pathway, which promotes autophagy. Additionally, polyphenols such as resveratrol and curcumin have been shown to induce autophagy via activation of sirtuins (SIRT1), enzymes involved in cellular stress resistance and repair [194]. The activation of autophagy helps clear damaged mitochondria, reduce the accumulation of toxic metabolites, and promote myocardial recovery, thereby mitigating the long-term cardiotoxic effects of chemotherapy. A low glycemic index diet promotes a healthier gut microbiome by supporting the growth of beneficial bacteria that help metabolize complex carbohydrates and produce short-chain fatty acids (SCFAs) [195]. These SCFAs have anti-inflammatory and antioxidant properties, contributing to the protection of cardiovascular and renal health.

By reducing the intake of rapidly digestible carbohydrates, a low glycemic index diet prevents the overgrowth of pathogenic bacteria that produce toxic metabolites; this, in turn, supports a healthier gut barrier function, reducing systemic inflammation and kidney injury [196]. The preservation of a balanced microbiome is particularly important in cancer patients, as dysbiosis has been linked to immune dysfunction, making the gut a critical factor in managing therapy-related complications. In summary, an anti-inflammatory and low-GI diet has multifaceted effects that contribute to the reduction of cardiotoxicity induced by anticancer drugs in patients with CKM syndrome [197]. Through the reduction of inflammation, oxidative stress insulin sensitivity, endothelial function, and autophagy, such a diet supports myocardial health and enhances cellular repair mechanisms, thereby potentially improving patient outcomes and reducing the risk of long-term cardiovascular complications. These dietary strategies not only provide a protective effect against drug-induced cardiotoxicity but also contribute to the overall management of the systemic metabolic dysregulation characteristic of CKM syndrome (Table 6).

## 7. Discussion

Cancer and cardiovascular disease (CVD) share a multitude of overlapping traditional risk factors, which contribute to their high co-prevalence. Epidemiological studies have demonstrated that hypertension, diabetes, dyslipidemia, obesity, smoking, and chronic inflammation significantly increase the risk of both malignancies and CVD. Obesity is a well-established risk factor for both cancer and CVD, with adiposity-driven insulin resistance, chronic low-grade inflammation, and endothelial dysfunction playing central roles. Excess visceral fat promotes tumorigenesis through increased insulin-like growth factor-1 (IGF-1) signaling and pro-inflammatory cytokines (IL-6, TNF-α), while also accelerating atherosclerosis and heart failure. Moreover, hyperglycemia and insulin resistance contribute to oxidative stress, endothelial dysfunction, and hypercoagulability, promoting both tumor growth and atherosclerotic plaque instability. Furthermore, dyslipidemia—characterized by high LDL cholesterol and triglycerides—drives atherogenesis and has been implicated in cancer cell proliferation via altered lipid metabolism. Chronic hypertension, mediated by endothelial dysfunction and increased arterial stiffness, exacerbates both CVD and cancer progression. Angiotensin II, a key regulator of blood pressure, has been shown to stimulate tumor angiogenesis and metastasis, highlighting the interplay between vascular and oncogenic pathways. Importantly, tobacco exposure remains a major modifiable risk factor for both diseases, contributing to DNA damage, increased oxidative stress, and inflammation. Chronic systemic inflammation, a hallmark of both conditions, fosters a tumor-permissive microenvironment while simultaneously driving vascular injury and atherosclerosis. Given these shared risk factors, recent clinical guidelines advocate for integrated cardio-oncology risk assessment models to identify high-risk individuals early in the disease trajectory.

Beyond shared risk factors, cancer progression and its therapies exert profound effects on cardiovascular health, contributing to long-term morbidity and mortality. Recent epidemiological data confirm that CVD has surpassed second malignancies as the leading cause of non-cancer death in cancer patients, particularly among survivors of breast, prostate, and hematologic cancers.

Certain malignancies, particularly hematologic cancers, induce a hypercoagulable state, increasing the risk of venous thromboembolism (VTE), myocardial infarction, and stroke. Tumor-secreted pro-inflammatory cytokines, such as IL-1β and IL-6, exacerbate endothelial dysfunction and vascular inflammation, further predisposing patients to CVD. Moreover, anticancer therapies could directly and indirectly expose patients to CVD. As is well known, anthracyclines (e.g., doxorubicin) are known for their dose-dependent cardiotoxicity, leading to heart failure via mitochondrial dysfunction and oxidative stress. Moreover, ICIs, involving anti-PD-1, anti-CTLA-4, anti OX40, and others, have been associated with immune-mediated myocarditis, pericarditis, and accelerated atherosclerosis. Tyrosine kinase inhibitors (TKIs) and VEGF inhibitors, commonly used in targeted therapy, contribute to hypertension, thromboembolic events, and endothelial dysfunction. Moreover, radiation therapy, particularly when directed at the chest (e.g., for breast cancer or Hodgkin lymphoma), induces coronary artery disease, valvular heart disease, and pericardial fibrosis, increasing long-term cardiovascular risk. Importantly, also endocrine therapies, such as aromatase inhibitors and androgen deprivation therapy, promote metabolic dysregulation, contributing to obesity, insulin resistance, and an increased risk of CVD.

Given these significant cardiovascular risks, cardio-oncology programs have emerged to optimize cardiac surveillance and preventive strategies in cancer patients. These programs focus on early risk stratification, biomarker-guided cardiotoxicity detection, and pharmacological interventions (e.g., beta-blockers, ACE inhibitors, SGLT2 inhibitors) to mitigate cardiovascular damage.

Recent clinical and epidemiological evidence has underscored the intricate interplay between cancer, CKM syndrome, and cardiovascular disease. CKM syndrome, a convergence of cardiac, renal, and metabolic dysfunction, frequently coexists in cancer patients, partly due to shared pathophysiological mechanisms such as chronic inflammation, oxidative stress, and dysregulated metabolic pathways [198]. This review critically examines the prevalence of CKM syndrome in cancer populations and explores the biochemical mechanisms linking this syndrome to an increased risk of MACE and atherosclerosis. Cancer therapies, particularly anthracyclines and tyrosine kinase inhibitors, are known to have off-target effects that impair cardiac and renal function, thereby aggravating metabolic imbalances [199]. These adverse effects are compounded in patients who already present with CKM syndrome, resulting in a higher prevalence within this subgroup. Chronic inflammation in CKM is characterized by high pro-inflammatory cytokines (e.g., IL-1β, IL-6, TNF-α), which activate NF-κB signaling. NF-κB upregulation leads to further cytokine synthesis and endothelial activation [200]. Moreover, cytokine-induced endothelial dysfunction results in increased vascular permeability, expression of adhesion molecules (VCAM-1, ICAM-1), and reduced NO bioavailability, thereby promoting a pro-atherogenic state [201]. Impaired mitochondrial function dysregulates cellular energy homeostasis; the resultant bioenergetic deficit and accumulation of damaged mitochondria initiate maladaptive autophagy responses, further impairing cardiomyocytes viability and endothelial function. Notably, insulin resistance, a hallmark of metabolic syndrome, elevates circulating insulin and alters lipid metabolism. High insulin levels activate signaling cascades (e.g., mTOR, PI3K/Akt) that can contribute to vascular smooth muscle proliferation and atheroma formation [202]. Moreover, hyperglycemia facilitates the non-enzymatic glycation of proteins, forming AGEs that bind to receptors (RAGE) on endothelial cells. This interaction triggers pro-inflammatory and pro-thrombotic cascades, intensifying vascular injury.

As is well known, the NF-κB and mitogen-activated protein kinase (MAPK) pathways are key integrators of inflammatory and stress responses [203]. In CKM syndrome, a persistent NF-κB activation not only promotes the transcription of pro-inflammatory genes but also upregulates MMPs, which degrade extracellular matrix components, destabilizing atherosclerotic plaques. Moreover, the MAPK pathway, through p38 MAPK and JNK, induces oxidative stress and cytokine signaling, further amplifying inflammation and apoptotic signaling in cardiac tissues [204].

Notably, through pharmacological and non-pharmacological strategies, involving SGLT2i, PCSK9i, GLP1-RA, and anti-inflammatory and low GI diet (Table 4), myocardial pAMPK activation and reduction of NLRP3/IL1 signaling could be achieved with associated cardioprotective effects [205]; these pathways are able to enhance glucose uptake, promoting fatty acid oxidation and inhibiting inflammatory responses. SGLT2i such as empagliflozin and dapagliflozin reduce heart failure hospitalization, chronic kidney disease progression, MACE, blood pressure, and body weight by activating AMPK/mTOR pathways, inhibiting NHE3 and RAAS, and reducing oxidative stress through enhanced glucose and sodium excretion, thereby alleviating cardiac and renal stress, improving mitochondrial efficiency, and decreasing fibrosis and inflammation. PCSK9i including inclisiran, evolocumab, and alirocumab lower LDL cholesterol, slow atherosclerosis progression, reduce major adverse cardiovascular events, and diminish inflammatory markers by promoting LDL receptor recycling and modulating anti-inflammatory pathways such as NF-κB and IL-6, effectively blocking PCSK9-mediated degradation of LDL receptors to enhance LDL clearance and reduce vascular inflammation [206]. GLP-1 receptor agonists like semaglutide and liraglutide contribute to weight loss, decreased atherosclerosis, lower major adverse cardiovascular events, blood pressure, and chronic kidney disease progression by engaging cAMP/PKA, PI3K/AKT, and AMPK pathways, inhibiting NF-κB to reduce inflammation, and enhancing endothelial nitric oxide production, which in turn increases insulin secretion, reduces appetite, improves endothelial function, and optimizes lipid metabolism [207]; soluble guanylate cyclase activators such as vericiguat and riociguat improve endothelial function, reduce heart failure exacerbations, and lessen fibrosis by targeting the NO-cGMP-PKG pathway with anti-fibrotic and anti-inflammatory effects that enhance vasodilation, decrease vascular stiffness, and improve cardiac and renal function [208]; and dietary interventions including mediterranean diet, low GI and plant-based diets lower obesity, blood pressure, atherosclerosis, insulin resistance, and CKD risk through activation of PPAR and AMPK, modulation of gut microbiota, reduction of TMAO production, and suppression of NF-κB and IL-6-mediated inflammation, collectively modifying lipid metabolism, reducing oxidative stress, and improving endothelial function [209]. Collectively, the increases in AMP-related signaling, the NHE3 and RAAS inhibition, oxidative stress reduction, the increases in LDL receptor recycling, as well as the activation in cAMP/PKA, PI3K/AKT, and NO-cGMP-PKG pathways achieved by SGLT2i, PCSK9i, GLP1RA, sGCa, and a proper low GI diet provide a set of preventive and therapeutic strategies in particularly vulnerable patients such as those with cancer and CKM, consequently reducing MACE events and improving OS [210].

Current management strategies for CKM syndrome rely on established guidelines from major cardiovascular, nephrology, and metabolic societies [211]. The American Heart Association (AHA) has emphasized the interconnected nature of cardiovascular disease, CKD, and metabolic dysfunction, advocating for an integrated approach that includes aggressive risk factor modification, lipid control, and glucose management [212]. The European Society of Cardiology (ESC) similarly highlights the importance of a holistic cardiovascular and metabolic risk assessment, incorporating lipid-lowering therapies (such as statins and PCSK9 inhibitors), glucose-lowering agents with cardiorenal benefits (such as SGLT2 inhibitors and GLP-1 receptor agonists), and rigorous blood pressure control to mitigate cardiovascular complications [213]. Meanwhile, Kidney Disease: Improving Global Outcomes (KDIGO) guidelines stress individualized reno-protective strategies, including RAAS blockade (using ACE inhibitors or angiotensin receptor blockers) and SGLT2 inhibitors to reduce CKD progression and cardiovascular risk [214]. Despite these advancements, current guidelines do not specifically address the complexities of CKM syndrome in cancer patients, where the interplay between oncologic treatments and CKM pathophysiology necessitates a more tailored approach.

Cancer therapies such as immune checkpoint inhibitors, anthracyclines, and VEGF inhibitors exacerbate CKM-related complications, highlighting the need for multidisciplinary cardio-oncology teams to integrate CKM-specific risk stratification into oncology care. Future personalized strategies should consider cancer type, therapeutic regimens, and individual CKM burden, ensuring optimal balance between oncologic efficacy and cardiometabolic safety. Further clinical research is warranted to develop evidence-based, cancer-specific CKM management protocols that improve overall survival and quality of life in this vulnerable population.

In conclusion, an early diagnostic strategy of CKM syndrome should integrate three phases before, during, and after therapies, such as:

- Baseline Risk Stratification (Pre-Therapy): Involving the assessment of traditional risk factors (hypertension, diabetes, obesity, dyslipidemia), a proper screening for subclinical cardiac dysfunction (echocardiography, GLS strain imaging), and renal function and metabolic assessment (eGFR, albuminuria, fasting glucose, lipid profile).
- During Therapy: Involving surveillance for emerging CKM components, a regular cardiac biomarker evaluation (troponins, NT-proBNP), metabolic changes (HbA1c, insulin resistance through HOMA score, visceral fat, fatty liver index), and renal function monitoring (GFR decline >10% signals early nephropathy).
- Post-Therapy Follow-up: Identifying long-term risk through annual screening for cardiovascular, renal, and metabolic complications; imaging for latent cardiomyopathy (MRI, global longitudinal strain imaging); early intervention strategies (involving SGLT2 inhibitors, GLP-1 agonists, RAAS inhibitors, and others).

By incorporating this structured approach, we can achieve early detection and risk modification of CKM syndrome in cancer patients, reducing both short- and long-term morbidity. Therefore, we propose a three-tiered approach to integrate these findings into routine clinical practice in cardio-oncology:

- Risk-Based Stratification Models: Develop structured risk assessment algorithms integrating AI-driven predictive models to identify high-risk patients before, during, and after cancer therapy.
- CKM-Focused Therapeutic Interventions through:
- Pharmacological Strategies: SGLT2i and/or GLP-1 receptor agonists for cardio–renal–metabolic protection; statins or PCSK9i for cholesterol reduction; beta-blockers and RAAS inhibitors for cardioprotection.
- Dietary and Lifestyle Modifications: Implementing plant-based and Mediterranean diets at low glycemic and insulinemic indices (which have been shown to reduce CKM burden by ~30%) [215].
- Structured daily exercise interventions personalized to each cancer survivor.
- Cardio-Oncology Integration into Survivorship Clinics: Establish multidisciplinary CKM clinics in oncology centers; standardized follow-up plans for early detection of cardiovascular and renal complications; real-time digital monitoring (wearables, telemedicine) for high-risk patients.

These measures ensure that CKM syndrome is not just recognized but actively managed in daily cardio-oncology departments, enhancing patient outcomes and long-term survivorship care.

## 8. Conclusions

CKM syndrome is a significant yet under recognized condition in cancer patients, contributing to increased morbidity, reduced QoL, and lower OS. CKM syndrome arises from the interconnected pathophysiology of CVD, CKD, and metabolic dysfunction, which are further exacerbated by anticancer therapies. Notably, anthracyclines, ICIs, tyrosine kinase inhibitors, and hormonal therapies can accelerate CKM progression by inducing cardiotoxicity, nephrotoxicity, and metabolic diseases. To address these challenges, a multidisciplinary approach incorporating novel therapeutic strategies is essential. Emerging treatments include SGLT2i for their reno-cardiometabolic benefits, GLP1-RA due to their reduction of HF and CKD progression, PCSK9i to reduce ASCVD risk, and sGCa for endothelial and cardiac function improvements. Additionally, dietary and lifestyle interventions play a crucial role in reducing metabolic dysfunction and enhancing overall patient outcomes. Further clinical research is needed to optimize personalized strategies for CKM prevention and treatment in this high-risk population. In conclusion, by addressing this critical gap in care, we can profoundly improve the lives of countless cancer patients.

## Figures and Tables

**Table 1:** Description of clinical scenario, pathophysiology, and associated CVD risk level at different stages of CKM syndrome.

Stage	Clinical Characteristics	Pathophysiological Features	CVD Risk Level
Stage 0	No evidence of metabolic, renal, or cardiovascular dysfunction	Normal metabolic and hemodynamic function	Low
Stage 1	Central obesity with early metabolic alterations	Adipose tissue dysfunction, mild insulin resistance, low-grade inflammation	Borderline
Stage 2	Established metabolic syndrome and/or early kidney dysfunction	Hypertension, dyslipidemia, hyperglycemia, increased renal stress	Intermediate
Stage 3	Subclinical cardiovascular disease with metabolic and/or renal impairment	Endothelial dysfunction, left ventricular remodeling, increased arterial stiffness	High
Stage 4	Overt cardiovascular disease with advanced metabolic and renal dysfunction	Atherosclerosis, heart failure, chronic kidney disease progression	Very High

**Table 2:** Search strategy used in Medline and EMBASE.

Database	Search String
Medline	“CKM AND cardiovascular diseases” OR “CKM AND cancer” OR “(CKM OR Metabolic Syndrome OR obesity) AND cardiology” OR “(CKM OR Metabolic Syndrome OR obesity) AND cardio-oncology” OR “(SGLT2i OR PCSK9i OR SGCa OR GLP-1 receptor agonist OR diet) AND CKM”
EMBASE	“CKM AND cardiovascular diseases” OR “CKM AND cancer” OR “(CKM OR Metabolic Syndrome OR obesity) AND cardiology” OR “(CKM OR Metabolic Syndrome OR obesity) AND cardio-oncology” OR “(SGLT2i OR PCSK9i OR SGCa OR GLP-1 receptor agonist OR diet) AND CKM”

**Table 3:** Different clinical pictures in cancer patients with CKM syndrome and thrapeutic challenges.

Clincal Aspect	Clinical Impact in Cancer Patients
Cardiovascular (CV) Risk	Increased risk of heart failure, arrhythmias, and atherosclerosis due to shared risk factors (hypertension, diabetes, obesity).
Kidney Dysfunction	Cancer therapies (e.g., chemotherapy, immunotherapy) may worsen CKD, leading to nephrotoxicity and increased mortality.
Metabolic Dysregulation	Insulin resistance, dyslipidemia, and obesity may accelerate cancer progression and worsen treatment response.
Cancer Therapy Toxicity	CKM syndrome exacerbates toxic effects of chemotherapy, targeted therapies, and immunotherapies, increasing MACE events.
Survival and Outcomes	Higher risk of treatment interruptions, complications, and reduced overall survival.
Inflammation and Immunity	Chronic inflammation (via metabolic and renal dysfunction) may promote cancer growth and reduce efficacy of immunotherapy.
Therapeutic Challenges	Need for multidisciplinary care; balancing cancer treatment with CV, renal, and metabolic management.

**Table 4:** Overall description of CV risk, kidney and metabolic dysfunction, cancer-related toxicity, survival, inflammation, and therapeutic challenges during CKM.

Biochemical Pathway	Cardiovascular Disease	Cancer	Relevance to CKM Syndromes	Key Signaling Molecules
Renin–Angiotensin–Aldosterone System (RAAS)	Hypertension, heart failure, atherosclerosis	Tumor angiogenesis, cancer cell proliferation	Dysregulated RAAS promotes endothelial dysfunction, fibrosis, and metabolic disturbances	Angiotensin II, AT1R, Aldosterone, ACE, Renin
Insulin Signaling Pathway	Insulin resistance, diabetic cardiomyopathy	Hyperinsulinemia-driven tumorigenesis (e.g., colorectal, breast cancer)	CKM syndrome involves metabolic syndrome, leading to hyperinsulinemia and inflammation	IRS-1/2, PI3K, AKT, mTOR, GLUT4
Inflammatory Pathways (NF-κB, IL-6, TNF-α)	Chronic inflammation, atherosclerosis, myocardial fibrosis	Tumor-associated inflammation, immune evasion	Systemic inflammation links CKM syndrome with endothelial dysfunction and fibrosis	NF-κB, IL-6, TNF-α, IL-1β, COX-2
Oxidative Stress Pathway (Nrf2, ROS, NOX)	Endothelial dysfunction, atherosclerosis	DNA damage, cancer progression	Oxidative stress accelerates CKM pathology via mitochondrial dysfunction and apoptosis	Nrf2, Keap1, ROS, NOX, SOD, GSH
Lipid Metabolism Pathway (PPAR, SREBP, LXR)	Dyslipidemia, atherosclerosis	Lipid-driven cancer proliferation (e.g., prostate, breast cancer)	CKM syndromes involve altered lipid metabolism, driving cardiovascular and oncogenic risks	PPAR-γ, SREBP-1, LXR, LDLR, HMGCR
AMPK/mTOR Pathway	Metabolic stress, heart failure, atherosclerosis	Cancer cell survival, metabolic adaptation	AMPK dysfunction in CKM syndromes leads to metabolic inflexibility and cardiovascular damage	AMPK, mTOR, ULK1, TSC2, Raptor
TGF-β/SMAD Pathway	Cardiac fibrosis, hypertrophy, kidney fibrosis	Epithelial–mesenchymal transition (EMT), metastasis	CKM-related organ fibrosis and metabolic dysfunction	TGF-β, SMAD2/3, SMAD7, α-SMA
HIF-1α Pathway	Hypoxia-induced vascular dysfunction	Hypoxia-driven tumorigenesis, angiogenesis	Hypoxia exacerbates CKM-related ischemic injury and metabolic imbalances	HIF-1α, VEGF, PHD2, VHL
Gut Microbiota and TMAO Pathway	Atherosclerosis, hypertension	Inflammation-driven carcinogenesis	Dysbiosis in CKM syndromes promotes metabolic endotoxemia and inflammation	TMAO, FMO3, LPS, SCFA
Uremic Toxins and Endothelial Dysfunction (Indoxyl Sulfate, p-Cresol)	Kidney injury, vascular calcification	Chronic inflammation, cancer promotion	CKM syndrome includes chronic kidney disease, exacerbating cardiovascular and oncogenic risks	Indoxyl sulfate, p-cresol, Klotho, eNOS

**Table 5:** Detailed overview of how anticancer therapies, involving hormonal therapies, anthracyclines, HER-2 blocking agents, VEGFi, and ICIs, influence the transition between CKM stages (from stage 0 to stage 4).

Therapy	Influence on CKM Progression	Stage Transition
Hormonal Therapies (AIs, anti-androgens)	Increase central obesity, insulin resistance, and dyslipidemia, promoting metabolic syndrome and early kidney dysfunction.	Stage 0 → Stage 1 → Stage 2
Doxorubicin (Anthracyclines)	Induces cardiotoxicity via oxidative stress, mitochondrial damage, and endothelial dysfunction, contributing to subclinical and overt cardiovascular disease.	Stage 2 → Stage 3 → Stage 4
HER2-Blocking Agents (Trastuzumab, Pertuzumab)	Dysregulate cardioprotective signaling, increasing the risk of heart failure, microvascular ischemia, and left ventricular dysfunction, particularly in patients with pre-existing CKM risk factors.	Stage 2 → Stage 3 → Stage 4
VEGF Inhibitors (Bevacizumab, Aflibercept) TKIs	Promote hypertension, endothelial dysfunction, arterial stiffness, and renal dysfunction, worsening metabolic and cardiovascular disease.	Stage 1 → Stage 2 → Stage 3 → Stage 4
Immune Checkpoint Inhibitors (anti-PD-1, anti-CTLA-4)	Exacerbate systemic inflammation, leading to myocarditis, endothelial dysfunction, and renal immune-mediated damage, accelerating CKM progression.	Stage 2 → Stage 3 → Stage 4

**Table 6:** Overall description of clinical outcomes of SGLT2 inhibitors (SGLT2i) (e.g., empagliflozin, dapagliflozin), PCSK9 inhibitors (PCSK9i) (e.g., evolocumab, alirocumab), GLP-1 receptor agonists (GLP1-RA) (e.g., semaglutide, liraglutide), soluble guanylate cyclase (sGC) activators (e.g., vericiguat, riociguat) and diet (Mediterranean, low-carb, plant-based) in patients with/without cancer. MACE—major adverse cardiovascular events, AMPK—AMP-activated protein kinase, mTOR—mechanistic target of rapamycin, RAAS—renin–angiotensin–aldosterone system; NHE3—sodium–hydrogen exchanger 3; NF-κB—nuclear factor kappa B; cAMP/PKA—cyclic AMP/protein kinase A; PI3K/AKT—phosphoinositide 3-kinase/protein kinase B; cGMP/PKG—cyclic guanosine monophosphate/protein kinase G; PPAR—peroxisome proliferator-activated receptor; TMAO—trimethylamine N-oxide; (↑: increase; ↓ decrease).

Therapeutic Approach	Clinical Outcomes	Key Signaling Pathways Involved	Mechanisms of Action
SGLT2 Inhibitors (SGLT2i) (e.g., empagliflozin, dapagliflozin)	↓ Heart failure hospitalization, ↓ CKD progression, ↓ MACE (major adverse cardiovascular events), ↓ BP, ↓ Body weight	AMPK/mTOR, NHE3 inhibition, RAAS inhibition, oxidative stress reduction	Increases glucose and sodium excretion, reduces cardiac and renal stress, improves mitochondrial efficiency, reduces fibrosis and inflammation
PCSK9 Inhibitors (PCSK9i) (e.g., evolocumab, alirocumab)	↓ LDL cholesterol, ↓ Atherosclerosis progression, ↓ MACE, ↓ Inflammatory markers	LDL receptor recycling, anti-inflammatory pathways (NF-κB, IL-6)	Blocks PCSK9-mediated LDL receptor degradation, enhances LDL clearance, reduces vascular inflammation
GLP-1 Receptor Agonists (GLP1-RA) (e.g., semaglutide, liraglutide)	↓ Body weight, ↓ Atherosclerosis, ↓ MACE, ↓ BP, ↓ CKD progression	cAMP/PKA, PI3K/AKT, AMPK activation, anti-inflammatory (NF-κB inhibition), endothelial NO production	Increases insulin secretion, reduces appetite, enhances endothelial function, reduces inflammation, improves lipid metabolism
Soluble Guanylate Cyclase (sGC) Activators (e.g., vericiguat, riociguat)	↓ Heart failure exacerbations, ↑ Endothelial function, ↓ Fibrosis	NO- cGMP/PKG pathway, anti-fibrotic, anti-inflammatory	Enhances vasodilation, reduces vascular stiffness, improves cardiac and renal function
Diet (Mediterranean, Low-Carb, Plant-Based)	↓ Obesity, ↓ BP, ↓ Atherosclerosis, ↓ Insulin resistance, ↓ CKD risk	PPAR activation, AMPK activation, gut microbiota–TMAO modulation, anti-inflammatory (NF-κB, IL-6 suppression)	Modifies lipid metabolism, reduces oxidative stress, improves endothelial function, modulates gut microbiota
